# Disuse plasticity limits spinal cord injury recovery

**DOI:** 10.1016/j.isci.2025.112180

**Published:** 2025-03-08

**Authors:** Kazuhito Morioka, Toshiki Tazoe, J. Russell Huie, Kentaro Hayakawa, Rentaro Okazaki, Cristian F. Guandique, Carlos A. Almeida, Jenny Haefeli, Makoto Hamanoue, Takashi Endoh, Sakae Tanaka, Jacqueline C. Bresnahan, Michael S. Beattie, Toru Ogata, Adam R. Ferguson

**Affiliations:** 1 Department of Neurological Surgery, Weill Institute for Neurosciences, Brain and Spinal Injury Center (BASIC), University of California, San Francisco (UCSF), San Francisco, CA, USA; 2 Department of Rehabilitation for the Movement Functions, Research Institute, National Rehabilitation Center for Persons with Disabilities, Saitama, Japan; 3 Department of Orthopaedic Surgery, Orthopaedic Trauma Institute (OTI), University of California, San Francisco (UCSF), San Francisco, CA, USA; 4 Neural Prosthesis Project, Department of Brain and Neuroscience, Tokyo Metropolitan Institute of Medical Science, Tokyo, Japan; 5 Department of Orthopaedic Surgery, Sensory and Motor System Medicine, Graduate School of Medicine, The University of Tokyo, Tokyo, Japan; 6 Department of Orthopaedic and Spine Surgery, Tokyo Metropolitan Geriatric Hospital, Tokyo, Japan; 7 Department of Orthopaedic Surgery, Saitama Red Cross Hospital, Saitama, Japan; 8 Department of Physiology, Advanced Medical Research Center, Toho University School of Medicine, Tokyo, Japan; 9 Faculty of Development and Education, Uekusa Gakuen University, Chiba, Japan; 10 Department of Rehabilitation Medicine, The University of Tokyo Hospital, Tokyo, Japan; 11 San Francisco Veterans Affairs Healthcare System (SFVAHCS), San Francisco, CA, USA

**Keywords:** Biological sciences, Neuroscience, Machine learning

## Abstract

Use-dependent plasticity after spinal cord injury (SCI) enhances neuromotor function, however, the optimal timing to initiate rehabilitation remains controversial. To test impacts of early disuse, we established a rodent model of transient hindlimb suspension in acute phase SCI. Early disuse in the first 2-week after SCI undermined recovery on open-field locomotion, kinematics, and swim tests even after 6-week of normal gravity reloading. Early disuse produced chronic spinal circuit hyper-excitability in H-reflex and interlimb reflex tests. Quantitative synaptoneurosome analysis of lumboventral spinal cords revealed shifts in AMPA receptor (AMPAR) subunit GluA1 localization and serine 881 phosphorylation, reflecting enduring synaptic memories of early disuse stored in the spinal cord. Automated confocal analysis of motoneurons revealed persistent shifts toward GluA2-lacking, calcium-permeable AMPARs in disuse subjects. Unsupervised machine learning associated multidimensional synaptic changes with persistent recovery deficits in SCI. The results argue for early aggressive rehabilitation to prevent disuse plasticity that limits SCI recovery.

## Highlights

• Early disuse undermines long-term locomotor recovery after spinal cord injury
• Early disuse induces chronic hyperexcitability of spinal reflexes after injury
• Early disuse overdrives GluA2-lacking AMPA receptors in chronic spinal cord injury
• Maladaptive spinal plasticity due to disuse hinders sustained recovery after injury

## Graphical abstract

**Figure:**
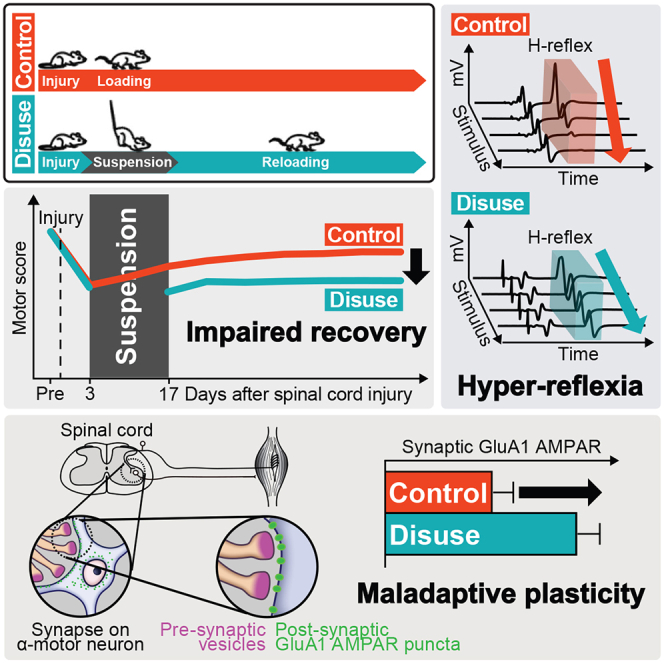

## Introduction

Neuroplasticity of spinal cord circuitry is a primary therapeutic target for promoting adaptive locomotor functions after spinal cord injury (SCI) and related disorders.^1^^,^^2^^,^^3^^,^^4^ However, not all neuroplasticity provides positive impacts on recovery. Spinal cord synaptic plasticity contributes to maladaptive changes, including central neuropathic pain and spasticity.^5^^,^^6^^,^^7^ This highlights the need to better understand conditions that shape plasticity after SCI^8^ to promote adaptive recovery while limiting maladaptive spinal cord changes.

Activity-dependent training has been reported to facilitate adaptive forms of neuroplasticity that advance recovery while mitigating maladaptive forms of neuroplasticity, such as hyper-reflexia/spasticity and central pain in both animal models and humans.^9^^,^^10^^,^^11^^,^^12^^,^^13^^,^^14^^,^^15^^,^^16^ Partial body weight-supported treadmill training improves ambulatory capability by promoting adaptive neuroplasticity within reorganized and residual neural networks following injury.^17^^,^^18^^,^^19^ Yet non-ambulatory input such as stand training or non-specific nociceptive input impairs recovery of stepping, providing evidence for task-specificity of neuroplasticity after SCI in both animal models and humans.^20^^,^^21^^,^^22^^,^^23^^,^^24^^,^^25^ In addition, transgenic alteration of muscle spindle feedback below incomplete SCI has a devastating impact on spontaneous recovery,^26^ suggesting that specific muscle load patterning modulates spinal cord circuitry.^27^ However, conflicting reports have called into question whether task-specific training can reliably impact recovery after SCI,^28^^,^^29^ and there is an ongoing debate about the role of cutaneous and loading afferents in shaping the recovery of function.

Most of this prior work has focused on augmenting input, but relatively little work has looked at the impact of inactivity and limb disuse in shaping subsequent recovery potential. In severe SCI patients, long-term limb immobilization is common. It has been hypothesized that limb inactivity contributes to the development of spasticity, intractable central pain, and nociceptive withdrawal reflexes in chronic SCI.^30^ In addition, limb immobilization induces transient neurological dysfunction in uninjured individuals, suggesting that inactivity may directly impact spinal cord circuitry,^31^^,^^32^ an effect that is likely to impact the recovery of function after SCI.^33^^,^^34^^,^^35^^,^^36^ Furthermore, biological mechanisms by which limb disuse after SCI alters spinal locomotor networks have yet to be clarified.

We hypothesized that early limb suspension after SCI would produce persistent maladaptive spinal cord neuroplasticity. We developed a “trained disuse” animal model of contusive thoracic SCI, adapting a model used to study microgravity tail suspension. Outcome measures included behavioral assessments, neurophysiological assessments, biochemical, and confocal analysis, and data integration using applied data science tools. Unsupervised machine learning related synaptic mechanisms of early disuse-induced maladaptive neuroplasticity to reduced locomotor recovery and reflex hyper-excitability in chronic SCI. Our findings highlight the importance of appropriately tuning early afferent input after SCI to prevent disuse-induced maladaptive neuroplasticity.

## Results

To test the impact of disuse-induced neuroplasticity, we established a novel “trained disuse” animal model in which animals were randomized after SCI to early disuse (2 weeks of suspension at 3–17 days post-injury, then hindlimb-reloading until 8 weeks post-injury), delayed disuse (2 weeks of suspension at 45 days post-injury, then until 11 weeks post-injury), or normal-loading control throughout the entire period of recovery after SCI.

### Randomization check

Randomization to disuse condition was checked against biomarkers of the injury severity, biomechanical injury parameters, baseline post-injury locomotor performance, and stress hormones. No significant group differences were observed for impact parameters, including actual force, displacement, and velocity (Figures S1A–S1C). There were no pre-randomization differences in plasma levels of pNF-H (Figure S1D) and BBB open-field locomotor scale (Figure S1E). Plasma corticosterone concentration peaked until 3 days post-injury (Figure S1F), followed by a time-dependent decrease returning to baseline 10 days post-suspension (13 days post-injury), with no significant difference produced by disuse (Figure S2). Together, the results demonstrate successful randomization.

### Early disuse impairs chronic locomotor recovery after spinal cord injury

**Figure 1:**
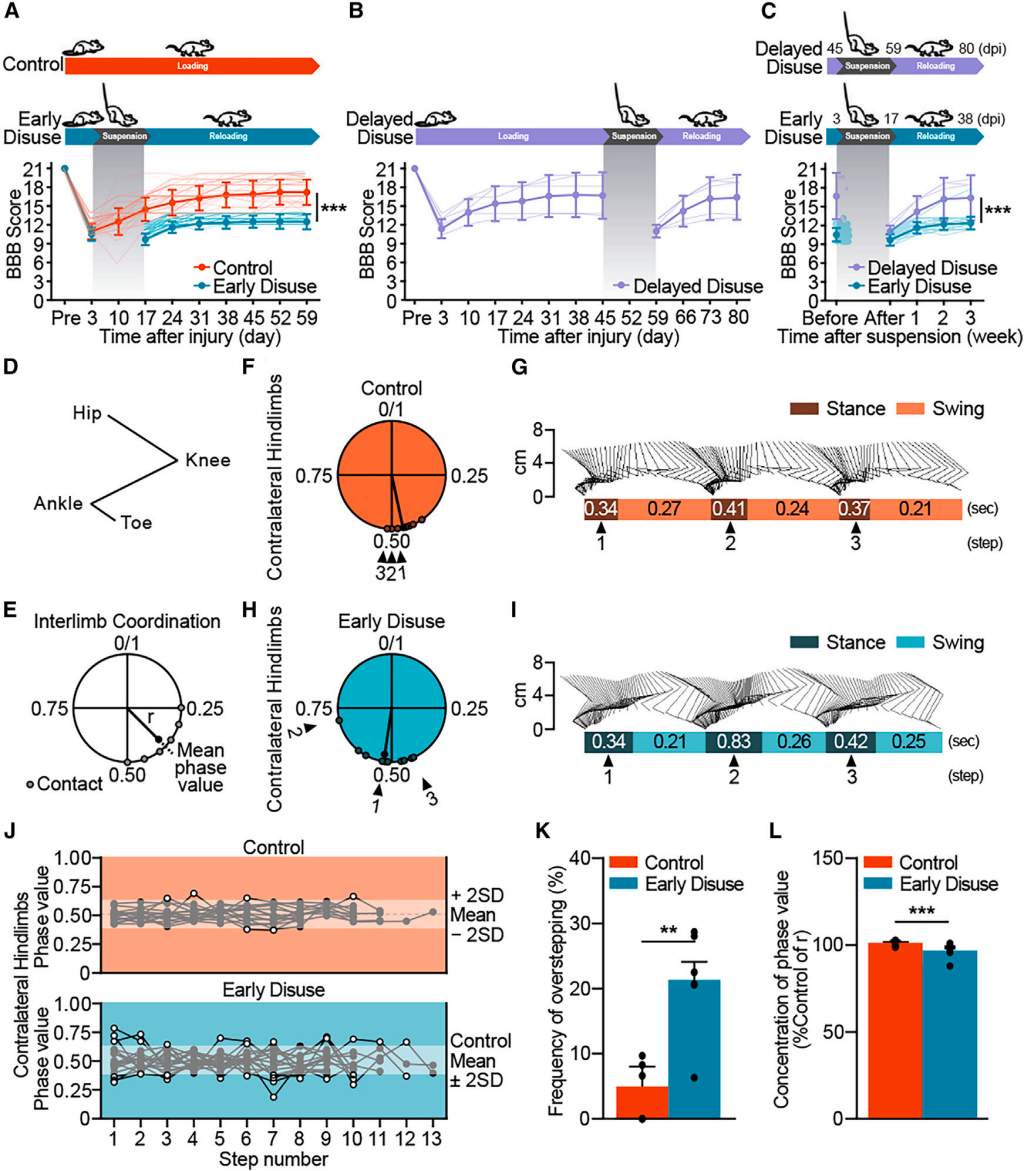
Early disuse impairs locomotor recovery after spinal cord injury (A and B) The experimental timeline and the BBB open-field locomotor scoring of disuse after spinal cord injury (SCI) among three groups: Early disuse (normal loading at 0–3 days post-injury, suspension at 3–17 days post-injury, reloading at 17–59 days post-injury; *n* = 63, cyan), Control (normal loading throughout the assessment; *n* = 54, orange), and Delayed disuse (normal loading at 0–45 days post-injury, suspension at 45–59 days post-injury, reloading at 59–80 days post-injury; *n* = 5, purple). (A) Early disuse produced chronically lower BBB recovery compared to control (effect of early disuse condition: *F*(1,115) = 307.729, ∗∗∗*p* = 2.6903E-34; Effect of Time: *F*(8,920) = 908.277, ∗∗∗*p* = 0.0E0; early disuse condition × time interaction: *F*(8,920) = 101.175, ∗∗∗*p* = 1.382E-120). Delayed disuse had a transient effect (B), and direct comparison of disuse groups time-aligned to the immediate post-suspension timepoints revealed higher BBB scores in the delayed disuse group compared to the early disuse group (C) (effect of early disuse condition: *F*(1,66) = 67.352, ∗∗∗*p* = 1.13E-11; effect of time: *F*(4,264) = 73.268, ∗∗∗*p* = 1.0949E-41; early disuse condition × time interaction: *F*(4,264) = 22.464, ∗∗∗*p* = 5.5576E-16). (D–L) To evaluate gait characteristics, the 3-D gait analysis was performed at 59 days post-injury (week 8 post-injury). (D) Anatomical landmarks of the hindlimb are represented by stick diagrams. (E) Schematic circular phase diagram of two-limb coordination. The circular plot indicates the timing of foot-ground contact during the swing phase of the reference limb within a step cycle that was assessed as the phase value from 0 to 1. The radius vector (r) value reflects the dispersion of interlimb coordination (0 equals to high dispersion), and the direction suggests phase variability. Representative circular phase diagrams of contralateral hindlimb coordination revealing irregular step cycles in the early disuse subject (Mean phase value = 0.526, r = 0.877) (H and I) marked by an increased contact phase of the step cycle and an increased phase dispersion of contralateral hindlimb coordination relative to the control subject (Mean phase value = 0.441, r = 0.959) (F and G). (J) The step sequence of the left hindlimb in all subjects of the early disuse group (lower panel, *n* = 6) and the control group (upper panel, *n* = 5) enabled comparison of the overstepping frequency (white point) (treadmill speed 16.6–25 cm/s, 8–13 consecutive foot contacts of hindlimb per trial, three trials per subject). (K) The frequency of overstepping compared with the range of mean ±2 standard deviation (SD) of the control group in the phase value represented a significant increase in the early disuse group (effect of early disuse condition: *F*(1,9) = 15.678, ∗∗*p* = 0.003; effect of trial: *F*(2,18) = 0.03, *p* = 0.971; early disuse condition × trial interaction: *F*(2,18) = 0.231, *p* = 0.796). (L) The concentration of the phase value reflecting the quantification of r compared to the control group showed a significant decrease in the early disuse group (effect of early disuse condition: *F*(1,9) = 28.177, ∗∗∗*p* = 4.89E-4; effect of trial: *F*(2,18) = 0.376, *p* = 0.692; early disuse condition × trial interaction: *F*(2,18) = 0.014, *p* = 0.986). The quantitative assessments of gait parameters were also performed regarding other types of interlimb coordination (Figure S4). ∗∗*p* < 0.01, ∗∗∗*p* < 0.001 by one-way or two-way analysis of variance (ANOVA). All data are shown as means ± standard error of the mean (SEM). Pre = pre-injury.

To test the impact of disuse on locomotor recovery after SCI, we performed analyses of both overground and treadmill gait using the BBB open-field locomotor scoring system and three-dimensional gait analysis. Early disuse produced long-term motor deficits that persisted into the chronic phase of ambulatory reloading (2–8 weeks post-injury) (Figure 1A). The BBB scores suggested that early disuse specifically impaired recovery of consistent forelimb-hindlimb coordination. Delayed disuse (6 weeks post-injury) did not have this effect; subjects demonstrated consistent coordinated forelimb-hindlimb stepping, indistinguishable from normal loading controls before suspension and showed not only a temporary function after disuse (Figures 1B and 1C). Evaluation of the gait characteristics by three-dimensional gait analysis demonstrated that early disuse generated a more irregular step cycle than the normal-loading controls caused by an increased contact phase of the step cycle as well as an increased phase dispersion of contralateral hindlimb coordination (Figures 1D–1I). The quantitative assessments of gait analysis indicate that the early disuse group produced a disruption of contralateral hindlimb coordination during treadmill gait (Figures 1J–1L and S4) consistently with the results of the open-field test (Figure 1A). Together, the results suggest that early limb disuse in SCI exacerbates long-term locomotor dysfunction, and these effects cannot be reversed simply with reloading exercises.

### Impact of early disuse after spinal cord injury on muscle atrophy

To test whether early disuse impacts muscle atrophy and recovery after SCI, we compared muscle mass of early disuse animals taken immediately after suspension (2 weeks post-injury) and after 6 weeks of reloading (8 weeks post-injury) with normal loading control after SCI at the endpoint assessment (8 weeks post-injury). Disuse of the lower hindlimb led to significant atrophy in the soleus muscles and gastrocnemius muscles in the early disuse group immediately after suspension (Figure S3). Such muscle changes have the potential to impact the interpretation of H-reflex testing by impacting the M-wave component of rate-dependent depression (RDD). However, muscle weight returned to normal after 6 weeks of reloading, suggesting that electrophysiological and behavioral recovery differences due to limb disuse cannot be explained by muscle atrophy alone.

### Early disuse increases spastic behavior during swimming in chronic spinal cord injury

**Figure 2:**
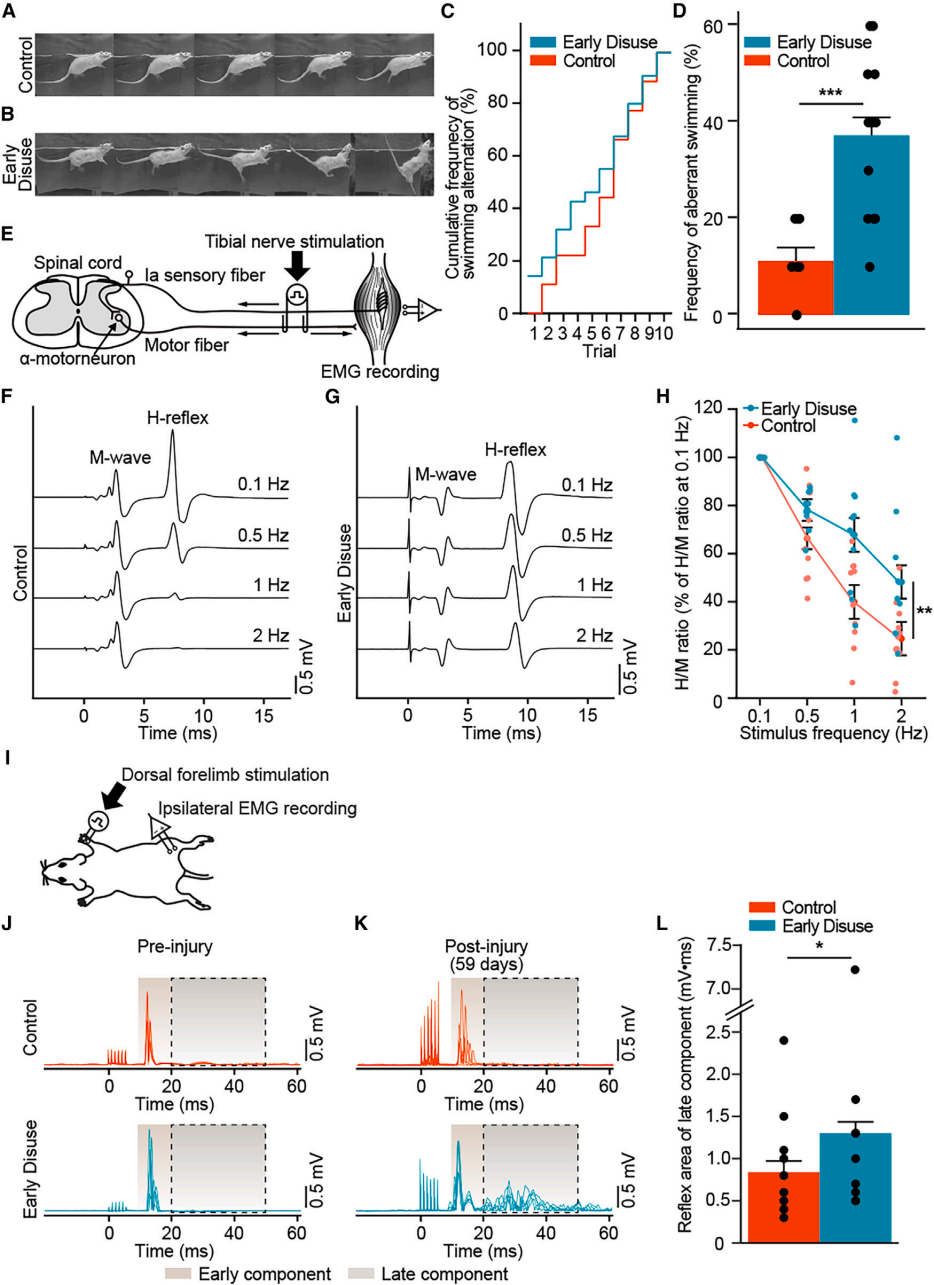
Early disuse produces spastic posture and hyper-excitability of spinal reflex circuits in chronic spinal cord injury (A and B) Representative sequential images of swimming behaviors in the subject of control (A) and early disuse (B) assessed at week 8 post-injury. (C) Cumulative distribution of the frequency of swimming alteration. The values were normalized to the number of trials showing aberrant posture during swimming in each group. Early disuse (*n* = 15) represented a significantly earlier trial of aberrational stretched hindlimb postures with unstable flexed trunk while swimming compared to control (*n* = 8) (Wilcoxon Signed-Rank Test, Z = −2.666, ∗∗*p* = 0.008). (D) The frequency of swimming alteration. The values were normalized to the number of all trials. The early disuse group demonstrated a significantly higher frequency of spastic posture events than the control group through all trials (effect of early disuse condition: *F*(1,21) = 20.817, ∗∗∗*p* = 1.69E-4; effect of trial: *F*(9.189) = 0.408, *p* = 0.93; early disuse condition × trial interaction: *F*(9.189) = 0.545, *p* = 0.84). (E) The Hoffmann’s reflex (H-reflex) testing setup. The subject of control (F) and early disuse (G) demonstrated the rate-dependent depression (RDD) of the H-wave through frequency (0.1, 0.5, 1, and 2 Hz) at week 8 post-injury. (H) The H-reflex amplitude at each stimulus frequency in both groups (each *n* = 10) was normalized to 0.1 Hz in the same group. The ratio of the H-reflex amplitude and corresponding M-wave amplitude was calculated (H/M ratio). M-wave responses had no significant difference across stimulus frequencies (Figure S5), whereas the RDD of the H-reflex was significantly diminished in the early disuse group compared to the control group (effect of early disuse condition: *F*(1,18) = 7.01, ∗*p* = 0.016; effect of stimulation frequency: *F*(3,54) = 93.212, ∗∗∗*p* = 2.42E-21; effect of early disuse condition × stimulation frequency interaction: *F*(3,54) = 4.97, ∗∗*p* = 0.004). (I) The interlimb reflex testing paradigm. (J) In pre-injury, both groups (each *n* = 9) demonstrated no differences at the late component (20–50 ms, gray) of the interlimb reflex, whereas early disuse increased the reflex response at week 8 post-injury (K). (L) Quantitation revealed a significant main effect of early disuse condition (effect of early disuse condition: *F*(1,16) = 6.122, ∗*p* = 0.025; effect of time: *F*(8,128) = 2.29, ∗*p* = 0.025) but not an early disuse condition × time interaction over weeks (*p* > 0.05) (Figure S6E). Non-significant effect and interaction on the early component (10–20 ms, brown) of the interlimb reflex (all *p* > 0.05) (Figures S6A–S6D). ∗*p* < 0.05, ∗∗*p* < 0.01, ∗∗∗*p* < 0.001 by one-way or two-way ANOVA. All data are shown as means ± SEM.

To further characterize motor deficits in suspension conditions after SCI, we performed a swimming test at 8 weeks post-injury.^37^^,^^38^^,^^39^^,^^40^^,^^41^ Early disuse subjects demonstrated aberrant posture characterized by a stretched hindlimb with an unstable, flexed trunk and/or marked tail extension during swimming (Figures 2A and 2B). The frequency of spastic postures in the early disuse group was significantly earlier (Figure 2C) and higher (Figure 2D) than the control group through all trials. This result suggests that early disuse after SCI induces altered posture, indicating spasticity that chronic ambulatory reloading is incapable of restoring.

### Early disuse induces chronic reflex hyper-excitability after spinal cord injury

To further investigate spasticity/hyper-reflexia in segmental spinal cord circuits in early disuse after SCI, we performed the H-reflex testing as well as the interlimb reflex testing. The H-reflex testing at 8 weeks post-injury demonstrated the conventional pattern of the “M-wave” that is produced by a directly activating α-motorneurons followed by the delayed “H-wave” produced by Ia afferent neuron activation of α-motorneurons monosynaptically by the stimulation (Figures 2E–2G and S5). The RDD of the H-reflex is taken as a normal response, and failure of H-reflex suppression is taken as a measure of hyper-reflexia/spasticity index in SCI.^42^^,^^43^ The control group demonstrated pronounced RDD with increasing frequency, whereas the early disuse group exhibited significantly less RDD than the control group (Figures 2F–2H). We also tested the interlimb reflex recorded in the medial gastrocnemius muscle with the forepaw electrical stimulation (Figure 2I). Both groups demonstrated no differences in the early component (Figures S6A and S6D) or the late component (Figures 2J, S6B, and S6E) of the interlimb reflex in pre- and post-injury (Figure S6C), but a substantial increase was seen in the late component after early disuse that persisted even after 6 weeks of normal loading (Figures 2K, 2L, and S6E). Taken together, the results indicate that early disuse after SCI induces a persistent hyper-excitability of spinal reflex circuits.

### Early disuse induces persistent changes in glutamatergic synaptic receptors after spinal cord injury

**Figure 3:**
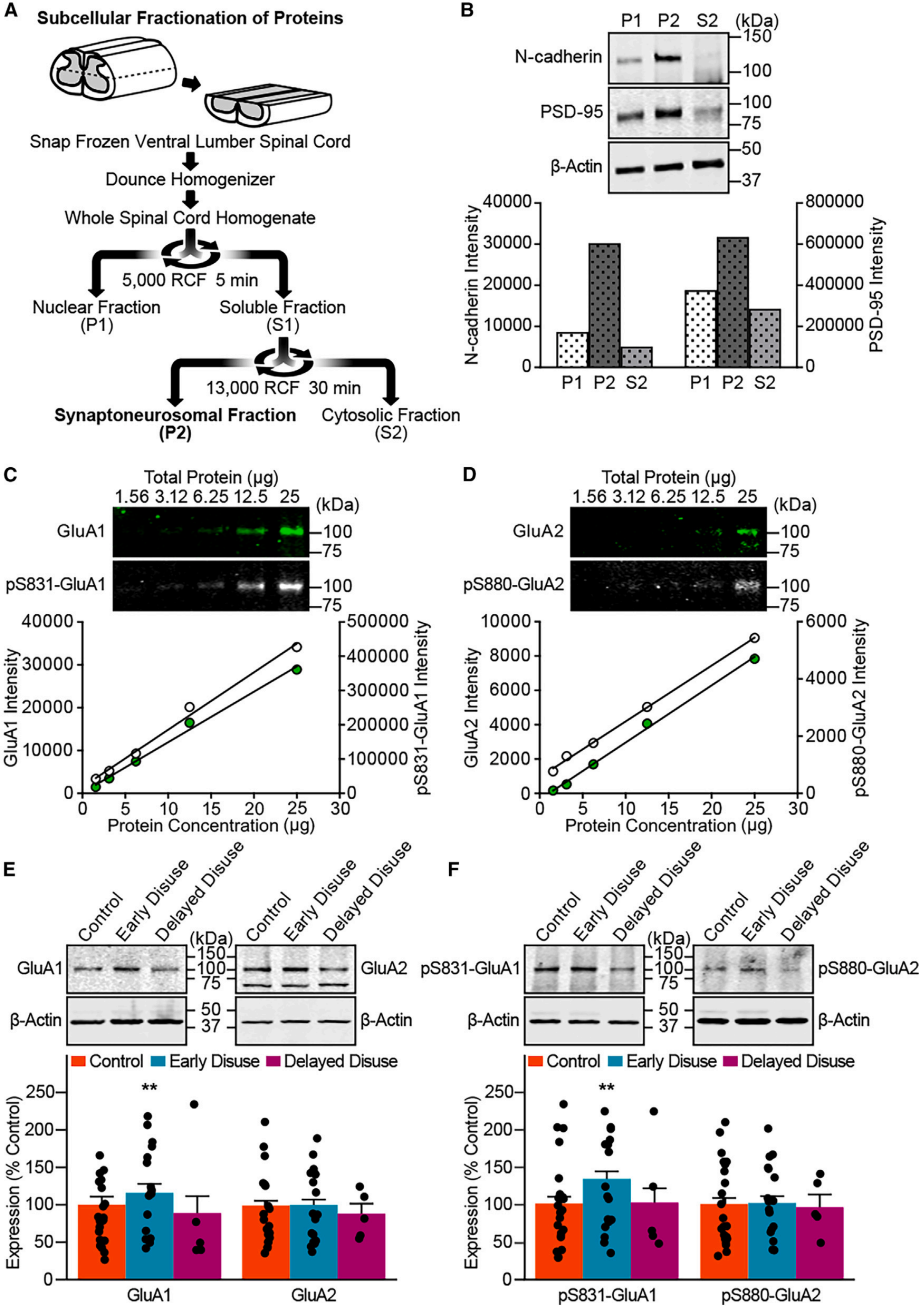
Early disuse chronically shifts GluA2-lacking phospho-AMPAR localization to synapses To assess the impact of early suspension after SCI on the level of excitatory neurotransmitter receptors in the ventral spinal cord, quantitative near-infrared western blot analysis was performed in early disuse (*n* = 18), control (*n* = 22), and delayed disuse (*n* = 5) at week 8 post-injury. (A) Subcellular fractionation diagram. Snap-frozen lumbar ventral spinal cord was biochemically processed to isolate the synaptoneurosome and membrane-enriched fractions (P2). (B) The P2 fraction was characterized by enrichment in the plasma membrane protein N-cadherin and modest PSD-95 enrichment, indicating synaptoneurosomal-enriched pellet fraction. (C) Glutamate AMPA receptors (AMPARs) are implicated in experience-dependent synaptic plasticity underlying learning and memory as excitatory neurotransmitter receptors.^47^^,^^48^^,^^49^ Quantitative linear range determination for GluA1 and its phosphorylation at PKC/CamKII site pS831 (pS831-GluA1) using a protein dilution curve revealed a wide linear detection range. (D) Linear range detection for GluA2 and its PKC/CamKII target pS880 (pS880-GluA2). Laser scanning intensities for each target protein were selected by the closest linear relationship between fluorescent intensity and total protein (all R^2^ > 0.99). Subsequent analyses were performed at each optimal scanning intensity to ensure fluorescence linearity. (E) Upper panel, representative western blot images for linear intensity quantification of GluA1 and GluA2. Lower panel, unbiased florescent densitometry of plasma membrane-enriched fractions of the ventral spinal cord revealed a significant main effect of early suspension condition on GluA1 (effect of early disuse condition: *F*(2,4.992) = 17.587, ∗∗*p* = 0.005), but no significant difference between early disuse conditions on GluA2 (effect of early disuse condition: *F*(2,3.996) = 0.434, *p* = 0.675). (F) Upper panel, representative western blot images for linear intensity quantification of pS831-GluA1 and pS880-GluA2. Lower panel, there was also a significant main effect of early suspension condition on pS831-GluA1 (effect of early disuse condition: *F*(2,3.697) = 29.06, ∗∗*p* = 0.005), but not pS880-GluA2 (effect of early disuse condition: *F*(2,3.947) = 0.079, *p* = 0.925). ∗∗*p* < 0.01 by one-way analysis of covariance (ANCOVA). All data are shown as means ± SEM. Full-length blots are shown in Figure S7.

To test for alterations that may contribute to suspension conditions induced chronic reflex hyper-excitability, we assayed the level of excitatory neurotransmitter receptors in ventral cord synapses in a dedicated sub-cohort of subjects from the early disuse, delayed disuse, and normal-loading control groups at 8 weeks post-injury. Ionotropic glutamate AMPA (α-amino-3-hydroxy-5-methyl-4-isoxazole propionic acid) receptors (AMPARs) are responsible for the majority of fast excitatory neurotransmission in the central nervous system, including the spinal cord.^44^ Trafficking of both AMPAR subunits GluA1 and GluA2 to and from synapses with their phosphorylation states (pS831-GluA1 and pS880-GluA2) play critical roles in multiple forms of rapid synaptic plasticity.^45^ In addition, GluA2-lacking AMPARs are uniquely calcium-permeable and inward-rectifying and drive long-term changes in plasticity by initiating intracellular calcium-dependent signaling cascades that result in further changes in plasticity.^46^ Biomolecular fractionation of the ventral spinal cord yielded successful enrichment of N-cadherin (neuronal membrane) and PSD-95 (synaptic membrane) in the second pellet fraction (P2) (Figures 3A and 3B). Each target protein was quantified by quantitative linear near-infrared laser scanning western blot analysis relative to a standardized protein dilution curve with laser settings optimized for the linear relationship between fluorescent intensity and total protein (Figures 3C and 3D). Early disuse produced a significant increase in GluA1 but not GluA2 (Figure 3E), consistent with increased calcium-permeable GluA2-lacking AMPARs (CP-AMPARs) in ventral cord synapses. In addition, there was an increase in gain-of-function GluA1 phosphorylation (pS831-GluA1; target of calcium-dependent kinases PKC and CamKII) with no change in GluA2 subunit at serine 880 (pS880-GluA2) compared to both delayed disuse and control subjects (Figure 3F). Together, the biochemistry results suggest that early disuse after SCI induces a persistent increase in CP-AMPARs that lasts into the chronic phase despite 6 weeks of reloading.

### Early disuse increases synaptic expression of calcium-permeable AMPARs in chronic spinal cord injury

**Figure 4:**
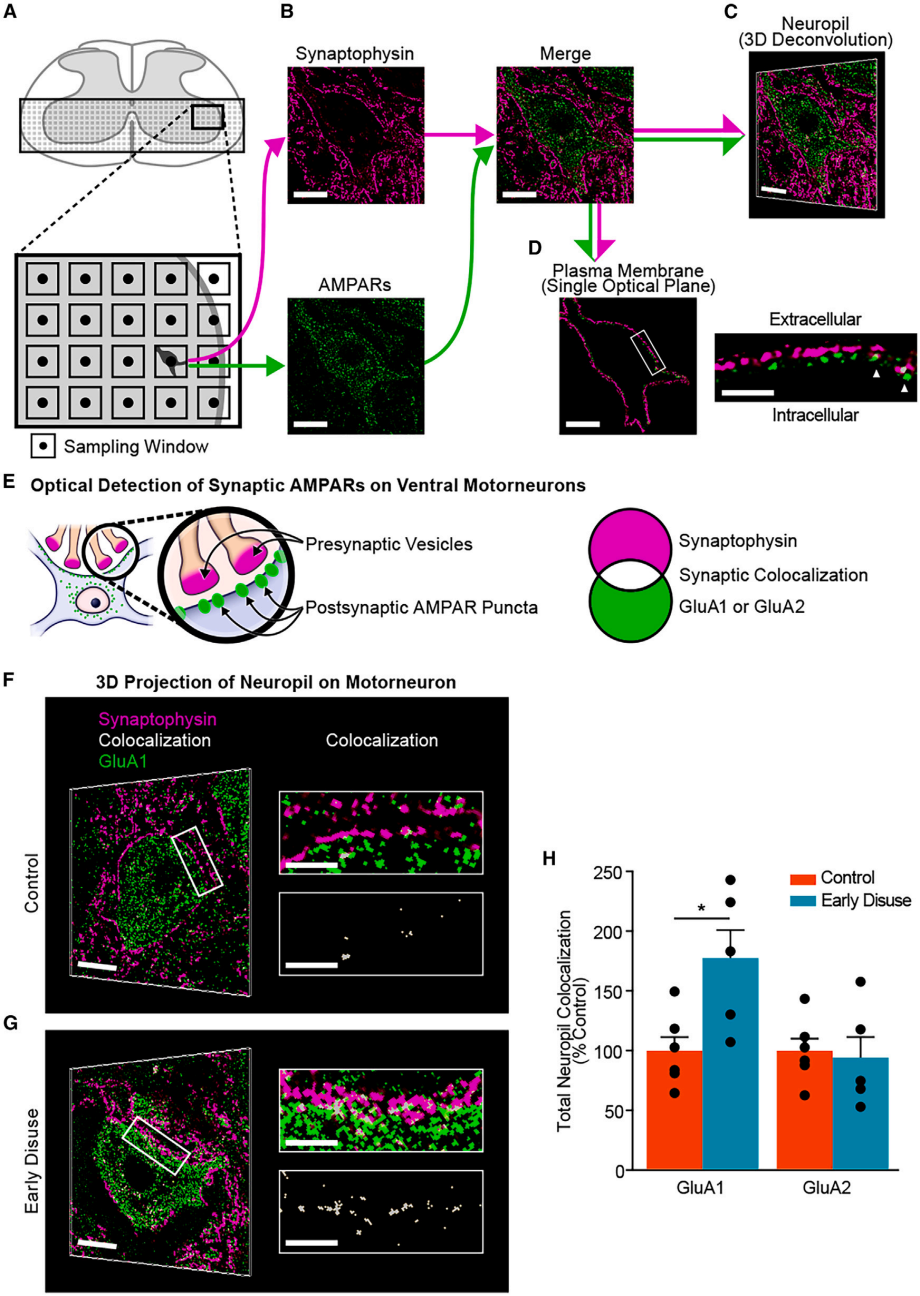
Early disuse persists synaptic expression of GluA2-lacking AMPARs as changes in synapse numbers on ventral horn neurons and dendritic neuropils after spinal cord injury Overdrive of glutamate AMPARs is known to reflect maladaptive spinal cord plasticity in central nervous system trauma.^12^^,^^50^ (A–E) Workflow diagram of the automated and unbiased high-resolution robotic confocal microscopy. To assess synaptic levels of glutamate AMPARs on dendritic fields and somata of large ventral horn neurons, the randomized microscopic detection and analysis were performed blindly by spinning disk confocal scan system at week 8 post-injury according to the established algorithm.^12^^,^^50^ (A) Fluorescently labeled large ventral horn neurons (diameter >40 μm) indicated presumptive motorneurons and were detected centrally in the sampling window (80 × 80 μm) at 63× magnification. (B) A stack of high-resolution images was taken in the z-plane through a 650 nm filter for presynaptic synaptophysin (upper panel) and a 490 nm filter for postsynaptic AMPARs (lower panel) at each level separately (scale bar: 20 μm). (C) Scanned confocal z stacks were deblurred by 3-D blind iterative deconvolution, and then the total expression of synaptic colocalized AMPAR puncta was quantified using the established approach. (D) Single optical planes showing maximal synaptic colocalization of presynaptic synaptophysin and postsynaptic AMPARs on the somata were selected among the z stacks, and then the optical fraction images of the somatic membrane were generated to assess the synaptic AMPAR expression on the plasma membrane (left panel). Representative optical detection of the presynaptic vesicle, postsynaptic AMPAR subunit, and synaptic colocalization (white arrows) are shown in the enlarged image of the boxed region from the plasma membrane image (right panel; see Figure 5). (E) Schematic overview on synaptic colocalization of AMPAR subunits GluA1 and GluA2. The overlapping of presynaptic synaptophysin (magenta) and synaptic AMPAR subunit (GluA1 or GluA2; green) puncta indicates synaptic colocalization (white). (F) Representative merged 3-D confocal image of large ventral horn neurons showed postsynaptic GluA1 (green) and synaptophysin (magenta) positive presynaptic terminals on the soma with surrounding dendritic neuropils in the control subject (left panel). The enlarged image of the boxed region from (F, left panel) demonstrates relatively few synaptic colocalized GluA1 puncta (lower right panel, white) within the merge image (upper right panel) in the control subject. (G) Representative merged motorneuron from the early disuse subject demonstrating greater numbers of membrane and dendritic GluA1 colocalized to the synapses (left panel). The enlargement of the boxed region from (G, left panel) demonstrates high levels of synaptic colocalized GluA1 puncta (lower right panel, white) within the merge image (upper right panel) in the early disuse subject. Representative images of synaptic GluA2 puncta are shown in Figure S8. (H) Quantification of synaptic colocalized AMPAR expression with synaptophysin though the confocal z stacks in the early disuse group (*n* = 5; total 164 cells, total 11,971 optical planes for GluA1; total 138 cells, total 9,788 planes for GluA2) compared to the control group (*n* = 6; total 163 cells, total 13,037 images for GluA1; total 156 cells, total 10,894 optical planes for GluA2) on the somata and dendritic neuropil. Random effects ANOVA controlling for non-independence of within-subject and within-section variability confirmed that early disuse significantly increased in synaptic colocalization of GluA1 (effect of early disuse condition: *F*(1,9) = 8.05, ∗*p* = 0.019) but no significant difference in synaptic colocalization of GluA2 (effect of early disuse condition: *F*(1,9) = 0.074, *p* = 0.792). There was a non-significantly difference between both groups in the presynaptic synaptophysin and postsynaptic AMPAR subunit (Figure S8). Representative examples reflect the group median. ∗*p* < 0.05 by one-way ANOVA. All data are shown as means ± SEM. Scale bars in the lower magnification images represent 20 μm, and the scale bar in the higher magnification image represents 5 μm.

To test whether early disuse directly impacted synaptic AMPAR expression at the single-cell level of spinal motorneurons, we developed a robotic confocal data collection protocol, coupling spinning disk microscopy with systematic automated sampling, 3-D blind iterative deconvolution and a software-based image analysis pipeline yielding super-resolution images. This high-resolution and high-content image analysis allows quantifying synaptic localization of AMPARs sterically as optical colocalization of post-synaptic AMPARs and pre-synaptic synaptophysin in the three-dimensional synaptic fields on large ventral horn motorneurons surrounding dendrites (Figures 4A–4E). Prior work has shown these methods to be sensitive to the rapid trafficking of CP-AMPARs to spinal cord synapses.^12^^,^^50^^,^^51^ We evaluated synaptic levels of AMPARs on large ventral horn motorneurons of the early disuse group compared to the normal-loading control group at 8 weeks post-injury. Quantitative analysis revealed a significant increase in synaptic colocalized GluA1 on the somata of motorneuron with three-dimensional volume dendritic neuropil in the early disuse group (Figures 4G and 4H) compared to the control group (Figure 4F). There was also an increase in the total number of synaptophysin, but no significant difference in total synaptic GluA1 puncta in early disuse relative to control (Figure S8). Synaptic colocalized GluA2 (Figure 4H) and total synaptic GluA2 (Figure S8) did not change significantly. As a chronic expression of both GluA1 and GluA2/3 are decreased in lumbar ventral horn motorneurons of moderate contusive thoracic injury,^52^ this result indicates the pathological state that early disuse after SCI induces persistent synaptic trafficking of CP-AMPARs on large ventral horn neurons.

### Early disuse alters CP-AMPAR localization on motorneurons in chronic spinal cord injury

**Figure 5:**
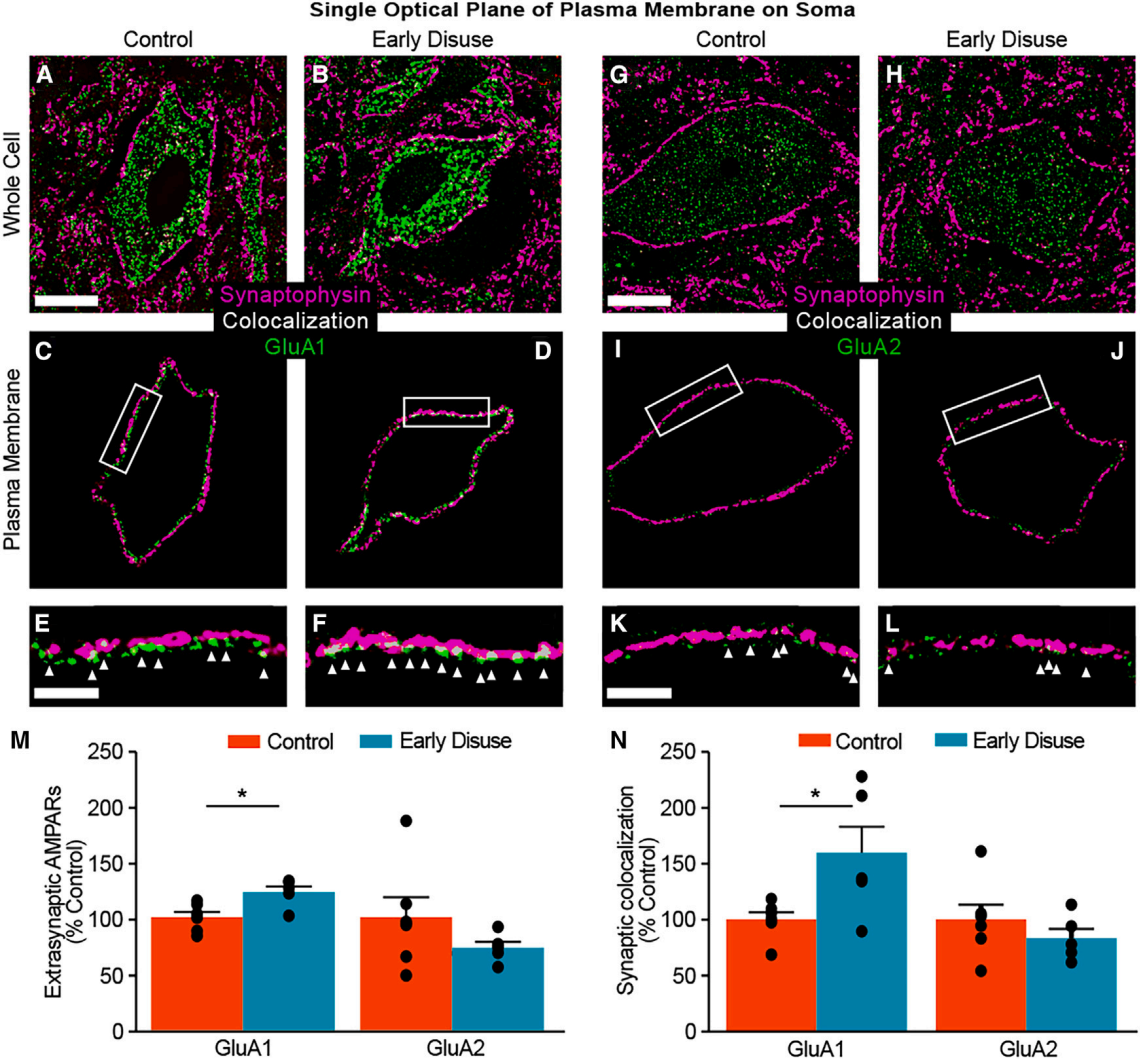
Early disuse induces chronic changes in extrasynaptic and synaptic expression of GluA2-lacking AMPARs on the plasma membrane of large ventral horn neurons after spinal cord injury (A–L) Activity-dependent synaptic plasticity depends on the long-lasting enhancement in the strength of excitatory synaptic transmission underlying AMPA trafficking from the intracellular endosomes to the non-synaptic (extrasynaptic) and synaptic sites in CNS.^44^^,^^50^^,^^53^^,^^54^ To assess activity-dependent long-term changes of extrasynaptic and synaptic AMPAR expression on the plasma membrane of large ventral horn neurons, the single optical planes showing maximal synaptic colocalization of presynaptic synaptophysin and postsynaptic AMPARs were selected for each motorneuron z stack in the early disuse group (*n* = 5) and the control group (*n* = 6) at week 8 post-injury using the established algorithm (see Figures 4A–4E).^50^ Analysis of representative merged single optical planes with the highest colocalization showed synaptophysin (magenta) and AMPAR subunit (green) on the soma with surrounding dendrites in the control subject (A GluA1; G GluA2) and the early disuse subject (B GluA1; H GluA2). Representative plasma membrane images were created by blind-to-condition tracing of the cell-boundary to generate a 2 μm width optical fraction of somatic membrane in the control subject (C GluA1; I GluA2) and the early disuse subject (D GluA1; J GluA2) (scale bar: 20 μm). The enlarged images of the boxed region from the above plasma membrane images demonstrate differential localization of extrasynaptic and synaptic AMPAR subunit in the control subject (E GluA1; K GluA2) and the early disuse subject (F GluA1; L GluA2). White arrows indicate synaptic colocalization of AMPAR puncta. Single optical plane analysis revealed high levels of synaptic colocalized GluA1 puncta tightly in early disuse (F), whereas relatively few synaptic colocalized GluA2 puncta in both subjects (K and L). (M) Quantification of AMPAR expression at extrasynaptic sites on the plasma membrane of large ventral horn neurons indicated presumptive motorneurons. Extrasynaptic GluA1 was significantly increased in the early disuse group (effect of early disuse condition: *F*(1,9) = 8.662, ∗*p* = 0.016) and a decrease in extra-synaptic GluA2 but no significance (effect of early disuse condition: *F*(1,9) = 1.482, *p* = 0.254). (N) Quantification of colocalized AMPAR expression with synaptophysin at synaptic sites on the plasma membrane of large ventral horn neurons. Synaptic colocalization of GluA1 was significantly increased in the early disuse group (effect of early disuse condition: *F*(1,9) = 5.903, ∗*p* = 0.038), whereas synaptic GluA2 showed no significant difference (effect of early disuse condition: *F*(1,9) = 0.865, *p* = 0.377). Representative examples reflect the group median. ∗*p* < 0.05 by one-way ANOVA. All data are shown as means ± SEM. Scale bars in the lower magnification images represent 20 μm (A and G), and the scale bar in the higher magnification image represents 5 μm (E and K).

Post-synaptic AMPARs are located at both synapses as well as non-synaptic (extra-synaptic) sites on the somata with surrounding dendrites. Extrasynaptic sites have been shown to play a critical role in modulating the reserve pool of AMPARs for rapid changes such as long-term potentiation (LTP) and homeostatic synaptic scaling in CNS.^44^^,^^47^^,^^48^ The optical fraction of somatic membrane was generated from the single optical planes of the large ventral neuron with maximal synaptic colocalization by semi-automated microscopic extraction and analyzed to evaluate subcellular localization of post-synaptic AMPARs on the motorneurons blindly (Figures 4D and 5A–5F for GluA1; Figures 5G–5L for GluA2). Automated quantification revealed a significant increase in extrasynaptic GluA1 and a decrease in extrasynaptic GluA2 but no significant difference in the somatic membrane of large ventral neurons in the early disuse group relative to the control group (Figure 5M). Early disuse also significantly increased synaptic colocalized GluA1 relative to control, whereas GluA2 demonstrated non-significant differences (Figure 5N). Altogether, the biomolecular and confocal data suggest that early disuse after SCI induces persistent excitatory synaptic transmission of CP-AMPARs and GluA2 removal from extra-synaptic sites on large ventral horn motor neurons.

### Machine learning links chronic impairments to synaptic plasticity produced by early disuse

**Figure 6:**
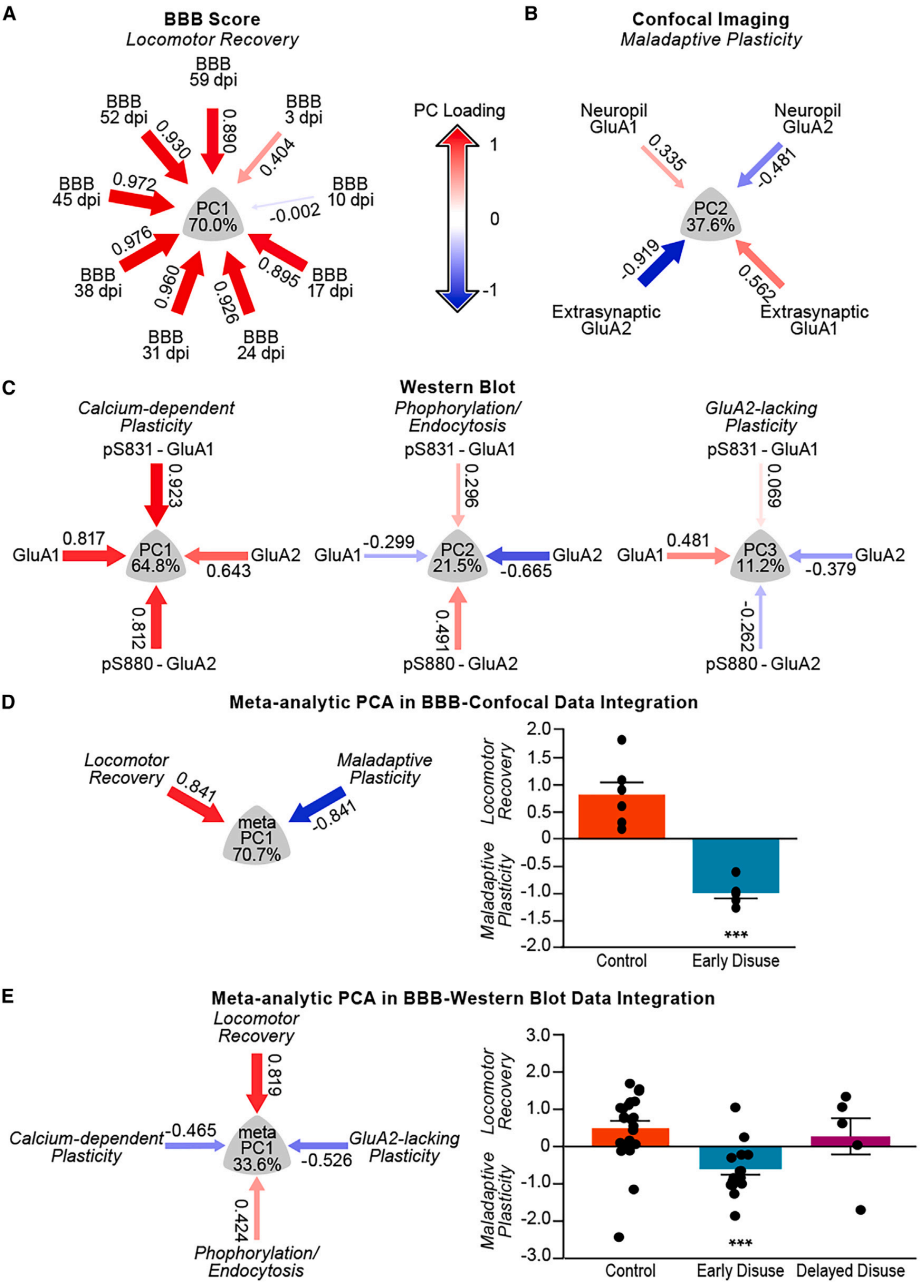
The robust multidimensional interactions between AMPAR mediated-maladaptive synaptic plasticity and impaired locomotor recovery in chronic spinal cord injury To identify the association between time-dependent locomotor recovery and chronic AMPAR over-drive on spinal motorneurons, nonlinear principal component analysis (NLPCA) was performed using the manifold learning approach. (A) PC loading pattern of locomotor recovery by the BBB open-field locomotor scoring system accounted for 70.0% of total variance with high loading on PC1 outcome representing NLPCA of “locomotor recovery”. (B) PC loading pattern of chronic AMPAR over-drive on spinal motorneurons by confocal microscopy accounted for 37.6% of total variance with higher synaptic GluA1 and lower GluA2 on PC2 outcome representing NLPCA of “maladaptive plasticity”. (C) PC loading pattern of chronic AMPAR synaptic membrane localization and gain-of-function phosphorylation by western blot accounted for 11.2% of total variance with higher GluA1 with lower GluA2 and the PKC/CamKII phosphorylation sites on PC3 outcome representing NLPCA of “GluA2-lacking AMPAR plasticity” (right panel). The NLPCA for western blot also revealed PC1 reflecting “calcium-dependent plasticity” (left panel, 64.8% of total variance) and PC2 reflecting “phosphorylation/endocytosis” (middle panel, 21.5% of total variance). (D) Meta-analytic PCA in BBB-Confocal data integration (metaPCA_Imaging_) of “locomotor recovery” and “maladaptive plasticity” PC scores supported the hypothesis that maladaptive glutamatergic synaptic transmission on motorneurons predicts the extent of locomotor impairment plasticity on spinal motor neurons (left panel, 70.7% of total variance) and early disuse condition performs worse than the control condition (right panel, effect of early disuse condition: *F*(1,9) = 39.5, ∗∗∗*p* = 0.000143). (E) Meta-analytic PCA in BBB-western Blot data integration (metaPCA_Biochem_) of “locomotor recovery” and 3 western blot PC scores supports the hypothesis that overdrive of AMPAR synaptic plasticity is inversely related to locomotor recovery (left panel, 33.6% of total variance) and early disuse condition performs worse on this axis than delayed disuse and control conditions (right panel, effect of early disuse condition: *F*(2,42) = 9.43, ∗∗∗*p* = 0.000414). Meta-analytic PCA in BBB-neurophysiological data integration (metaPCA_H-reflex_; metaPCA_Interlimb reflex_) supported the hypothesis that chronic reflex hyper-excitability predicts persistent locomotor impairments (Figure S9). ∗∗∗*p* < 0.001 by one-way ANOVA. All data are shown as means ± SEM.

To identify the association between time-dependent locomotor recovery and chronic CP-AMPAR over-drive on spinal motorneurons, we harnessed the manifold learning approach of nonlinear principal component analysis (NLPCA). To test the interaction between locomotor recovery and AMPAR-mediated spinal cord plasticity, we began by running separate waves of NLPCA, one for each of the domains (locomotor recovery, confocal imaging, and western blot). The NLPCA for locomotor recovery revealed a first principal component that accounted for 70% of the total variance, with the BBB scores loading highly together onto the PCs with increasing magnitude over time, indicating that this PC represents “locomotor recovery” (Figure 6A). The NLPCA for confocal measures of the protein expression of extra-synaptic and synaptic AMPAR subunits revealed a first principal component that accounted for 37.6% of the total variance, and interestingly, showed GluA1 and GluA2 measures loading in the opposite direction, indicating that this PC is representative of a GluA2-lacking “maladaptive plasticity” (Figure 6B). The NLPCA for western blot measures of the protein expression of AMPAR subunits and the phosphorylation sites revealed 3 PCs that each captured orthogonal partitions of the variance reflecting “calcium-dependent Plasticity”, “phosphorylation/endocytosis”, and “GluA2-lacking AMPAR plasticity” (Figure 6C; PC1 64.8%, PC2 21.5%, PC3 11.2%, respectively). To assess the interaction between locomotor recovery and synaptic AMPAR levels, we ran a meta-analytic PCA (*meta*-PCA) on confocal data, synthesizing NLPCA findings of “locomotor recovery” and “maladaptive plasticity” (Figure 6D). This confocal *meta*-PCA revealed a significant inverse relationship, with locomotor recovery PC scores decreasing as maladaptive plasticity increased (Figure 6D, left panel). Confocal metaPC scores (metaPC_Imaging_) were then derived and used as endpoints to test the hypothesis that early disuse drives subjects along the maladaptive plasticity-recovery axis. Analyses revealed that the early disuse condition had significantly higher maladaptive plasticity and worse chronic locomotor recovery relative to the control condition (Figure 6D, right panel). We then applied *meta*-PCA to western blot synaptosome-recovery scores (metaPC_Biochem_) synthesizing NLPCA findings of “locomotor recovery” with “calcium-dependent Plasticity”, “phosphorylation/endocytosis”, and “GluA2-lacking AMPAR plasticity”, respectively. Analyses revealed that locomotor recovery loaded opposite the GluA2-lacking plasticity construct identified in western blot PC3 and calcium-dependent plasticity in western blot PC1. This loading pattern indicates that higher metaPC_Biochem_ scores reflect poorer locomotor recovery associated with greater maladaptive plasticity (Figure 6E, left panel). Statistical hypothesis testing on metaPC_Biochem_ revealed that early disuse conditions had greater maladaptive plasticity associated with worse recovery of function relative to delayed disuse and control conditions (Figure 6E, right panel). Both *meta*-PCA on H-reflex testing (metaPCA_H-reflex_) and interlimb reflex testing (metaPCA_Interlimb reflex_) also suggested that chronic reflex hyper-excitability was highly relevant to chronic locomotor impairments (Figure S9). Taken together, the machine learning analyses revealed the robust multidimensional association between overdriven plasticity in synaptic AMPARs, spinal reflex hyper-excitability as well as impaired locomotor function in chronic SCI.

## Discussion

The impact of acute limb disuse after SCI on subsequent recovery is poorly understood. We hypothesized that early suspension below SCI induces persistent maladaptive plasticity in the lumbar spinal cord that enhances spasticity and contributes to impairments in locomotor recovery. We harnessed a suspension model originally developed to simulate microgravity in spaceflight^55^^,^^56^ and found that limb disuse early after SCI (weeks 1–2 post-injury) led to chronic recovery impairments in open-field locomotor, gait analysis, and swimming outcomes relative to normal-gravity controls. Delayed disuse (6–8 weeks post-injury) did not have this adverse effect, suggesting a window of vulnerability in the acute post-injury phase.

Neurophysiological assessments (H-reflex; interlimb reflex) implicated chronic hyper-excitability in the segmental local reflex circuit, propriospinal pathway, and α-motorneuron pool as key mechanisms driving recovery impairments after early hindlimb disuse. Synaptoneurosomal protein assays provided a mechanism for the observed spinal hyperexcitability, revealing that early disuse drives gain-of-function phosphorylation of synaptic AMPARs at serine 831 (pS831) on the GluA1 subunit and increases CP-AMPARs in synapses. Automated spinning disk confocal imaging of an independent cohort of subjects confirmed that early disuse increases GluA1 but not GluA2 subunits in synapses on the membrane of large ventral motorneuron somata and surrounding dendritic fields, suggesting that chronic increases in GluA2-lacking AMPARs on motorneurons endure for at least 6 weeks after cessation of forced disuse. These effects were recapitulated at extrasynaptic sites on motorneuron somata, a membrane domain known to be the site of rapid endocytosis and exocytosis of AMPARs that regulate the pool of receptors for synaptic insertion.^57^ Together, the data strongly suggest that early limb disuse after SCI generates a maladaptive spinal cord “memory trace” in the ventral motor pools that endures into the chronic phase, even after returning the subject to normal hindlimb loading conditions.

Integrative machine learning analytics applied to the full set of AMPAR endpoints revealed a multidimensional maladaptive plasticity biosignature that predicted locomotor recovery in a graded fashion (i.e., the greater the maladaptive plasticity biosignature score, the worse the recovery). A second-order analysis generated meta-PC scores combining behavior and synaptic endpoints into a composite measure reflecting maladaptive plasticity biobehavioral outcome. Using this biobehavioral meta-PC composite score as a primary endpoint, we found a significant effect of early limb disuse on maladaptive biobehavioral neuromotor scores. The results strongly suggest that early disuse after SCI drives spinal cord maladaptive plasticity that impairs recovery of function into the chronic phase.

### Implications and relationship to prior work

Our application of a “trained disuse” model was designed to mimic the natural time course of recovery in clinical SCI subjects, which is characterized by acute phase bed rest followed by chronic phase ambulatory training in the rehab setting.^58^ The suspension model was first developed to study multi-organ “wasting” (cardiovascular and bone-density studies) in prolonged bed rest and space flight-induced microgravity conditions.^55^ In the present study, we repurposed this disuse model in the context of a mild contusive SCI^59^^,^^60^ designed to avoid ceiling and floor effects with respect to locomotor function for SCI for behavioral assessments. The suspension period was based on prior work demonstrating that 2 weeks is sufficient to alter locomotor performance by impacting anti-gravity soleus muscle function.^61^

The present data, together with prior work, suggests that the acute post-injury spinal cord is susceptible to aberrant peripheral sensory inputs following injury because descending brain pathways are compromised and unable to control spinal cord excitability.^62^ Thus, afferent input to the injured spinal cord has a strong potential to shape intrinsic spinal cord plasticity, impacting not only dorsal horn pain pathways, but also interneuron circuitry, and ventral horn motor pathways.^8^^,^^63^ Identification of the type and timing of aberrant afferent input is essential for optimizing rehabilitation to tip the balance between adaptive and maladaptive neuroplasticity. Rapid excitatory synaptic neurotransmission in the spinal cord has been well-studied as a driver of central pain syndromes.^64^ Nociceptive input in acute SCI has been found to impair locomotor recovery, suggesting that nociceptive sensitization generates a form of maladaptive plasticity that interferes with adaptive recovery.^21^^,^^65^^,^^66^^,^^67^^,^^68^^,^^69^^,^^70^^,^^71^^,^^72^ Much less is known about disuse-induced (passive) forms of maladaptive plasticity.^8^ Early limb immobilization restricts activity-dependent afferent input, but also provides aberrant proprioceptive sensory feedback to the spinal cord from atrophied muscle fibers,^34^^,^^35^^,^^36^ which may alter central spinal cord circuitry, thereby reducing adaptive retraining.^33^ On the other hand, early limb loading (rehabilitation) after SCI may protect against the development of maladaptive plasticity, as seen in the delayed disuse group in the present study.

Disuse by suspension in intact animals generally alters locomotion performance, an effect that is due, in part, to neuronal effects involving the modification of motorneuron excitability in lumbar spinal cord circuitry.^73^^,^^74^^,^^75^^,^^76^^,^^77^ Self-retraining in-cage after SCI allows spontaneous intrinsic recovery,^33^^,^^78^ and hindlimb ambulatory reloading has the potential to restore locomotor performance,^79^ soleus muscle activity,^73^^,^^79^ and motorneuron excitability of lumbar spinal cord circuitry^75^ within two weeks post-suspension in intact animals, mimicking rehabilitation of chronic SCI. The present findings suggest that the acute disuse has detrimental effects, limiting the impact of self-rehabilitation upon return to normal loading in the home cage.

Our findings suggest that disuse limits function by rapidly altering synaptic weights in the spinal cord by impacting systems involved in CNS memory formation. The canonical receptor system involved in forming CNS memories, the AMPAR subunit (GluA1-GluA4), mediates the vast majority of fast excitatory neurotransmission in the CNS, including the spinal cord.^80^ AMPARs are known to contribute to long-term lasting synaptic plasticity as well as modulation of AMPAR levels, and the phosphorylation site reflects activity-dependent synaptic plasticity, providing the substrate for maladaptive neuronal plasticity in the spinal cord.^64^^,^^81^^,^^82^ Our previous work identified that neuroinflammatory signaling cascades in acute SCI can drive post-synaptic localization of CP-AMPARs as well as alterations in gamma-aminobutyric acid receptor A (GABA-A) on ventral horn spinal neurons that together may shape pain, dysfunction, and excitotoxicity in contusive SCI.^12^^,^^83^ Nociception-induced aberrant afferent input into the spinal cord drives complex changes in excitatory tone, potentially flipping the balance of inhibition and excitation in non-linear ways. For example, GABAergic systems may become excitatory and contribute to maladaptive plasticity in the presence of nociceptive input below SCI.^84^^,^^85^^,^^86^ Nociceptive input also produces a synaptic signature with gain-of-function GluA1 S831 phosphorylation without any effects in GluA2 S880 phosphorylation on lumbar ventral horn motorneurons in the acute phase of complete thoracic transection SCI.^50^ Post-synaptic localization of CP-AMPARs is caused by a rapid receptor trafficking mechanism from the intracellular endoplasmic reticulum to the synaptic membrane of CNS neurons reflecting post-translational modifications.^45^ Although GluA2-containing calcium-impermeable AMPA receptors (CI-AMPARs) are the predominant population in the hippocampus under normal conditions, CP-AMPARs are implicated in the induction of multiple forms of synaptic plasticity and scaling as well as the modulation of synaptic strength during long-term potentiation (LTP) in the hippocampus,^44^^,^^87^^,^^88^ and the development of neuronal networks in the spinal cord^89^ which is critical for regulating CNS functions and maintaining homeostasis. Early administration of a selective CP-AMPAR antagonist after SCI may restore established nociception-induced maladaptive synaptic plasticity in the complete thoracic transection SCI animal models, suggesting that CP-AMPARs play a critical role in maladaptive synaptic plasticity involving homeostatic forms of synaptic plasticity.^50^ The present study builds on this prior work, demonstrating that passive disuse also overdrives AMPARs in the spinal cord motor system and links these effects to sustained locomotor dysfunction and impaired recovery. This expands the clinical implications from “avoidance of nociception” to “avoidance of limb disuse” early after SCI as the requirement for optimal recovery of function. In addition, the results point to the modulation of CP-AMPARs in the spinal cord as a novel preventive target for reducing spasticity and improving recovery of function in SCI.

### Limitations of the study

Further work is needed to examine how these effects relate to other modulatory systems that are known to impact spasticity, such as the GABAergic systems that have long been targets of pharmacological interventions such as baclofen pumps to treat post-SCI spasticity, with mixed success and potentially detrimental impact on motor recovery.^90^ In addition, further work is needed to understand interactions between postsynaptic AMPAR plasticity and presynaptic plasticity such as neurite sprouting morphology and alterations in vGlut1 and vGlut2 presynaptic terminals after SCI. As a whole, the results point to the potential for more precise combinatorial therapies for SCI involving early rehabilitation combined with therapeutics that modulate excitatory tone to minimize maladaptive plasticity and promote recovery.

## Resource availability

### Lead contact

Further information and requests for resources and reagents should be directed to and will be fulfilled by the lead contact, Adam R. Ferguson (adam.ferguson@ucsf.edu).

### Material availability

This study did not generate new unique materials and reagents.

### Data and code availability

• All data are available via a CC-BY license at the NIH-supported repository for Spinal Cord Injury data, the Open Data Commons for Spinal Cord Injury (odc-sci.org): Morioka K., Tazoe T., Huie J., Hayakawa K., Okazaki R., Guandique C., Almeida C., Haefeli J., Hamanoue M., Endoh T., Tanaka S., Bresnahan J., Beattie M., Ogata T., Ferguson A. (2022) Dataset for limb disuse, synaptic maladaptation, and recovery impairments in female rats with T9 contusion SCI. ODC-SCI:612 https://doi.org/10.34945/F52P4M.
• This paper does not report original code.
• The remaining data and information are available within the main text or supplemental information. Any additional information required to reanalyze the data reported in this paper is available from the lead contact upon request.

## Acknowledgments

The authors would like to thank Xiaokui Ma for contributions to histology and immunohistochemistry; Jeffrey A. Sacramento for contributions to behavioral assessment; Makiko Morioka Miwa for contributions to gait analysis; Lauren VanCitters, and Daniel Fong for help with image analysis; Jennifer Truong, and Cleopa Omondi for help with protein assays and western blotting; Karen-Amanda Irvine, and Richard B. Ferguson for editorial assistance; Anastasia V Keller, Abel Torres-Espín, and Austin Chou for valuable comments; Kurt Thorn, DeLaine Larsen, and the staff at the Nikon Imaging Center at UCSF for assistance with all confocal image collection. This work was supported by grants from the 10.13039/100000002National Institutes of Health (NIH; NS088475, NS106899, NS122888 to A.R.F.; AR066262 to Jeffrey C. Lotz/A.R.F./K.M.), U.S. Department of Veterans Affairs (VA; I01RX002245, I01RX002787 to A.R.F.), 10.13039/100008191Wings for Life Spinal Cord Research Foundation (WFLUS008/12, WFLUS006/14 to A.R.F.; WFLUS013/13 to K.M.), 10.13039/100005191Craig H. Neilsen Foundation (224308 to A.R.F.; 313739 to J.H.), NASA Human Research Program (NNX14AP25G to Alan R. Hargens/A.R.F.), 10.13039/100008734Mitsui Sumitomo Insurance Welfare Foundation (09-011 to K.M.) and 10.13039/501100001691Japan Society for the Promotion of Science (JSPS; 10.13039/501100001691KAKENHI grant 21800092, 23700659 to K.M.; 18H03141 to T.T.; 23300204 to T.O.).

## Author contributions

K.M., T.O., and A.R.F. conceived and designed the experiments; K.M., T.T., K.H., R.O., C.F.G., and T.E. executed the experiments and contributed reagents/materials/analysis tools; K.M., T.T., J.R.H., J.H., T.O., and A.R.F. analyzed the data; K.M., J.R.H., C.A.A., J.H., and A.R.F. curated the data, and designed and implemented the analysis; K.M., T.T., J.R.H., M.H., J.C.B., M.S.B., T.O., and A.R.F. contributed to the interpretation of the results; K.M., T.O., and A.R.F. wrote the manuscript; K.M., T.T., J.R.H., M.H., J.C.B., M.S.B., T.O., and A.R.F. edited the manuscript, with comments from all authors.

## Declaration of interests

The authors declare no competing interests.

## STAR★Methods

### Key resources table

**Table:** 

REAGENT or RESOURCE	SOURCE	IDENTIFIER
**Antibodies**		

Mouse Monoclonal Anti-N-cadherin	BD Biosciences	Cat# 610920; RRID: AB_2077527
Mouse Monoclonal Anti-PSD-95	Thermo Fisher Scientific	Cat# MA1-046; RRID: AB_2092361
Rabbit Monoclonal Anti-β-Actin	Cell Signaling Technology	Cat# 4970; RRID: AB_2223172
Rabbit Polyclonal Anti-GluA1	Millipore	Cat# AB1504; RRID: AB_2113602
Rabbit Polyclonal Anti-GluA2	Millipore	AB1768-25UG; RRID: AB_2247874
Rabbit Monoclonal Anti-pS831-GluA1	Millipore	Cat# 04-823; RRID: AB_1977218
Rabbit Polyclonal Anti-pS880-GluA2	Millipore	Cat# 07-294; RRID: AB_568822
Mouse Monoclonal Anti-β-Actin	BD Biosciences	Cat# 612657; RRID: AB_399901
Goat Anti-rabbit IRDye 680RD	LI-COR Biosciences	Cat# 925-68071; RRID: AB_2721181
Goat Anti-mouse IRDye 800CW	LI-COR Biosciences	Cat# 926-32210; RRID: AB_621842
Rabbit Monoclonal Anti-GluA1	Millipore	Cat# 04-855; RRID: AB_1977216
Mouse Monoclonal Anti-Synaptophysin	Millipore	Cat# MAB5258-50UG; RRID: AB_95187
Rabbit Polyclonal Anti-NeuN	Millipore	Cat# ABN78; RRID: AB_10807945
Mouse Monoclonal Anti-GFAP	Millipore	Cat# MAB360; RRID: AB_11212597
Rabbit Monoclonal Anti-Vimentin	Cell Signaling Technology	Cat# 5741; RRID: AB_10695459
Rabbit Polyclonal Anti-GAP43	Millipore	Cat# AB5220; RRID: AB_2107282
Goat Anti-Rabbit IgG Alexa Fluor 488	Thermo Fisher Scientific	Cat# A-11034; RRID: AB_2576217
Goat Anti-Mouse IgG Alexa Fluor 647	Thermo Fisher Scientific	Cat# A-21236; RRID: AB_2535805

**Chemicals, peptides, and recombinant proteins**		

Pentobarbital Sodium	Kyoritsu Seiyaku	Cat# 7231101
Chloral Hydrate	Sigma-Aldrich	Cat# 15307
cOmplete Mini Protease Inhibitor Cocktail	Roche Diagnostics	Cat# 04693159001
PhosSTOP Phosphatase Inhibitor Cocktail	Roche Diagnostics	Cat# PHOSS-RO
10-20% Precast Tris-HCl Polyacrylamide Gel	Bio-Rad Laboratories	Cat# 1611395
Precision Plus Protein Kaleidoscope	Bio-Rad Laboratories	Cat# 1610375
Odyssey Blocking Buffer	LI-COR Biosciences	Cat# 927-40000
Tissue-Tek O.C.T. Compound	Sakura Finetek	Cat# 4583
Vectashield Mounting Medium with DAPI	Vector Laboratories	Cat# H-1200-10

**Critical commercial assays**		

Phosphorylated Neurofilament H Human ELISA	BioVendor	Cat# RD191138300R
Corticosterone EIA	IDS	Cat# AC-14F1
BCA Protein Assay Kit	Pierce	Cat# 23225

**Deposited data**		

Raw data	This paper	https://doi.org/10.34945/F52P4M

**Experimental models: Organisms/strains**		

Sprague-Dawley Rat	Charles River Laboratories	RRID: MGI: 5651135

**Software and algorithms**		

Kinema Tracer System	Kissei Comtec	http://www.kinematracer.com/
Odyssey Application Software Version 3.0	LI-COR Biosciences	RRID: SCR_014579
NIS Elements Imaging Software	Nikon Instruments	RRID: SCR_014329; https://calm.ucsf.edu/hardware-and-software-image-analysis
ImageJ	NIH	RRID: SCR_003070
AutoQuant	Media Cybernetics	RRID: SCR_002465
MetaMorph Automation and Image Analysis Software	Molecular Devices	RRID: SCR_002368
Adobe Photoshop	Adobe	RRID: SCR_014199
SPSS Statistics version 26.0	IBM	RRID: SCR_016479
Graphpad Prism	Graphpad	RRID: SCR_002798
Adobe Illustrator	Adobe	RRID: SCR_010279

**Other**		

Infinite Horizon Impactor	Precision Systems and Instrumentation	IH-0400; https://psiimpactors.com/product/ih400/
Treadmill	Muromachi Kikai	MK-680; https://muromachi.com/en/archives/english/1936/
Biological Amplifier	Nihon Kohden	NEC Biotop 6R12; https://doi.org/10.1371/journal.pone.0171937
AD Converter	Cambridge Electronic Design Limited	Micro 1401; https://ced.co.uk/products/microwaveformio
Constant Current Stimulator	Digitimer	DS7A; https://www.digitimer.com/product/human-neurophysiology/peripheral-stimulators/ds7a-ds7ah-hv-current-stimulator/
Plate Reader	Tecan	GENios; https://www.tecan.com/customer-news/genios-an-innovative-detection-device-that-delivers-flexibility-to-the-molecular-biology-laboratory-2347
Infrared Imaging System	LI-COR Biosciences	Odyssey Infrared Imaging System; RRID: SCR_023765; https://doi.org/10.1038/s41598-024-70096-0
Cryostat	Leica Biosystems	CM1950; RRID: SCR_018061; https://www.leicabiosystems.com/us/histology-equipment/cryostats/leica-cm1950/
Inverted Microscope	Nikon Instruments	ECLIPSE Ti inverted microscope system; RRID: SCR_021242; https://calm.ucsf.edu/csu-22-spinning-disk-confocal
Spinning Disk Confocal Microscope	Yokogawa Electric Corporation	CSU-22; RRID: SCR_020907; https://calm.ucsf.edu/csu-22-spinning-disk-confocal

### Experimental model and study participant details

#### Animals

Adult female Sprague-Dawley rats (60-80 day-old, n = 122, 220-250 g, Charles River Laboratories Japan, Inc.) were used across all experiments, considering the occurrence of bladder complications that lead to kidney reflux and related morbidity/mortality. All animals were maintained in a temperature-controlled room (23°C) with a 12-hour light/dark cycle and were provided *ad libitum* access to food and water. All experiments were approved by the ethical committee of the National Rehabilitation Center for Persons with Disabilities. Subjects were randomized to conditions, and analyses were performed blind-to-condition.

#### Surgical procedures

Operating procedures were performed as described previously.^41^^,^^91^ Briefly, animals were deeply anesthetized with pentobarbital sodium (50 mg/kg, intraperitoneal injection, Kyoritsu Seiyaku, Tokyo, Japan) after induction of anesthesia using isoflurane (3% mixed with oxygen and air, 5 L/min). A laminectomy was performed at thoracic vertebra nine (T9), and mild (50 kdyn) contusive injury was delivered using the Infinite Horizon impactor device (Precision Systems and Instrumentation, LLC, Fairfax, VA). Incisions were closed, suturing in layers. No analgesics were administered beyond surgical anesthesia because analgesics interfere with the biomolecular mechanisms under study. Impact parameters are provided in Figure S1.

#### Suspension

Subjects were randomly assigned to three groups (early disuse, delayed disuse, and control). The suspension was applied adapting a model that has been widely used to study the effects of limb disuse in microgravity and bed rest (see Figure 1).^55^^,^^56^ Briefly, hindlimbs were elevated approximately 30° above horizontal using flexible orthopaedic tape wrapped gently around the proximal two-thirds of the tail, preventing ischemia, and the limb position was adjusted for the persistent suspension using a spring-loaded movable arm. The forelimbs were in full contact with the ground at all times, and subjects were allowed to ambulate around the entire range of a special cage freely. Disuse subjects were individually housed in suspension conditions for 24 hours per day for two weeks. Control subjects were individually housed in normal cages. After suspension (or normal-loading control), subjects were housed individually for three weeks, then housed in large cages in groups of four until all endpoint assessments.

#### Animal assignment and sample size estimation

We assigned all experimental animals to two or three groups for each experiment (Table S1) according to the experimental design (see method details) and determined an adequate sample size per experiment to detect a significant difference among groups (Table S2). All molecular samples collected throughout the duration of the study were used for the aggregated data-driven discovery using nonlinear principal component analysis (Figures 6 and S9).

### Method details

#### Experimental design, randomization, and blinding

The timeline of the experimental procedures is shown in Figure 1. Mild thoracic SCI subjects were screened to ensure that all subjects could perform body weight support stepping at three days post-injury (defined as BBB open-field score of >9 points) prior to randomization. Subjects not meeting the baseline level of function were excluded *a priori* from the study. Subjects randomized into the early disuse group (n = 63) were given 2 weeks of suspension in the special cage starting at 3 days post-injury followed by 6 weeks of ambulatory reloading in a normal cage. Subjects in the normal loading control group (n = 54) were given normal weight-bearing post-injury without tail suspension for 8 weeks in a normal cage. Subjects in the delayed disuse group (n = 5) were randomized at 45 days from the normal-loading control cohort with coordinated forelimb and hindlimb stepping (defined as BBB open-field score of >14 points) and crossed-over into suspension for 2 weeks in the special cage followed by 3 weeks of ambulatory reloading in a normal cage. The delayed disuse subjects were run in parallel with their own cohort of early disuse and control subjects with a balanced sample size to directly assess the impact of timing on recovery of function and correlated synaptic biology. All behavioral, kinematic, biomolecular, and imaging analyses were performed blind to experimental conditions. All statistical analysis was performed by statisticians uninvolved in the data collection. All data are available at the Open Data for Spinal Cord Injury website and https://doi.org/10.34945/F52P4M.

#### Validation of injury severity

To assess validation of the experimental animal model before suspension, biomarker quantification of the injury severity was performed using the measurement of plasma pNF-H level, and assessment of the stress response was performed using the measurement of plasma corticosterone concentration via tail vein bleeding at 3 days post-injury. Combination analyses with parameters of the IH impactor device and the BBB open-field score at 3 days post-injury are provided in Figure S1. In addition, systematic differences in lesion size across groups are derived from post-mortem histological analysis presented in Figure S10.

(1) Assay of pNF-H

Cytoskeletal structural protein phosphorylated high molecular weight neurofilament subunit (pNF-H) was quantified as a blood-based injury biomarker of injury as described previously.^91^^,^^92^ Briefly, blood samples were acquired from the tail vein in the early disuse group (n = 35) just before suspension and the control group (n = 23). Plasma samples were collected in heparinized ethylenediaminetetraacetic acid (EDTA) tubes and stored at −80°C and plasma isolated centrifugation immediately prior to assay. An enzyme-linked immunosorbent assay (ELISA) kit (Human Phosphorylated Neurofilament H ELISA; BioVendor, Modrice, Czech Republic) was used according to the manufacturer’s instructions. All samples were tested in duplicate, and the average value of each sample was calculated from the standard curve. Subjects that showed below the limit of detection (70 pg/mL), indicating light injuries, were excluded from the experiment. The levels on 3 days post-injury are provided in Figure S1.

(2) Assay of corticosterone

Plasma corticosterone concentration was used as a representative biomarker to test for a stress response to suspension after SCI.^93^ Blood samples were acquired from the tail vein in the early disuse group (n = 10) and the control group (n = 10) at 1, 3, 6, 10, 13, and 17 days post-injury (pre-injury, just before suspension, 3, 7, and 10 days post-suspension, and just after suspension), respectively. The collection was performed every evening to avoid circadian variations. Plasma samples were centrifuged and stored at −80°C in EDTA tubes until assessment. The assay was conducted using an enzyme immunoassay (EIA) kit (Corticosterone EIA; IDS, Boldon, UK) according to the manufacturer's instructions. All samples were tested in duplicate, and the mean value of each sample was calculated from the standard curve. Samples with levels below the lower limit of detection (0.55 ng/mL) were excluded from the analysis. The levels on 3 days post-injury are provided in Figure S1, and the longitudinal data is shown in Figure S2.

#### Muscle mass

To assess suspension-induced deconditioning, measurement of lower hindlimb muscle weights was performed in gastrocnemius, tibial anterior, and soleus muscles. All muscles were excised bilaterally after spinal cord extraction in the early disuse group just after suspension (17 days post-injury; n=5) and the endpoint assessment (59 days post-injury; n=28), and the control group at the endpoint assessment (n = 34). The wet weight of each lower hindlimb muscle was measured and was normalized by the body weight (Figure S3). The average longitudinal body weight is shown in Figure S11.

#### Endpoint assessment strategy

Outcome was evaluated using 7 types of assessments: ⅰ) BBB open-field scoring was performed on post-injury day 3 prior to randomization, and then weekly until sacrifice; ⅱ) Three-dimensional gait analysis was performed at the terminal timepoint for detailed evaluation of ambulation recovery during treadmill gait (Figures 1 and S4); ⅲ) Swimming test was used to assess non-loading motor function and spasticity at the terminal timepoint (Figure 2); ⅳ) H-reflex testing at the terminal timepoint (Figures 2 and S5) and interlimb reflex testing from pre-injury to the terminal timepoint (Figures 2 and S6) as a terminal procedure for measuring persistent motorneuron excitability; ⅴ) Biochemical analysis (subcellular fractionation, protein assay, SDS-PAGE, quantitative near-infrared immunoblotting, statistical densitometric analysis) was performed to assess excitatory neurotransmitter receptor level changes in ventral horn synaptoneurosomes at the terminal timepoint (Figures 3 and S7); ⅵ) Immunohistochemical analysis and unbiased robotic confocal analysis of perfusion-fixed tissue sections were performed for evaluation of persistent AMPAR-mediated synaptic plasticity in ventral horn motorneurons (Figures 4, 5, and S8) with lesion pathology of thoracic spinal cords (Figure S10) at the terminal timepoint; ⅶ) All endpoints were analyzed by statisticians uninvolved in data collection, at both the univariate and multivariate level (Figures 6 and S9). Each endpoint is described below in detail in its own section of the methods.

#### Open-field testing procedure

Hindlimb locomotor function was evaluated using the Basso, Beattie, and Bresnahan (BBB) locomotor scale^94^ in the early disuse group (n = 63), control group (n = 54), and the delayed disuse (n = 5) from pre-injury to the endpoint assessments starting 3 days post-injury and then once a week, including just before/after suspension. In accordance with the experimental design, subjects were excluded *a priori* from all assessments if they could not perform body weight supported stepping consistently (BBB <9 points) at 3 days post-injury before suspension or could not perform coordinated forelimb-hindlimb stepping consistently as the control group (BBB <14 points) at 45 days post-injury before suspension.

#### Gait analysis

Gait parameters of the early disuse group (n = 6) and the control group (n = 5) were assessed on a treadmill (MK-680; Muromachi Kikai Co., Ltd., Tokyo, Japan) using a three-dimensional motion analysis system (Kinema Tracer System; Kissei Comtec, Nagano, Japan) at the endpoint assessments. Four high-speed digital cameras, positioned to capture the same field of view orthogonally, were used to record the positioning of colored markers which were placed on the skin at five anatomical landmarks of the hindlimb bilaterally; the distal/lateral fifth metacarpophalangeal joint (MCP), the trochanter major (hip), the knee, the lateral malleolus (ankle), and the distal/lateral fifth metatarsophalangeal joint (MTP) as well as one anatomical landmark of forelimb at the distal/lateral fifth MCP bilaterally. Gait analysis data were collected at a sampling rate of 120 Hz, and the cameras were calibrated to collect data in a rectangular cube (5 × 20 × 10 cm) in a pre-determined frame within the treadmill runway. Treadmill speed was set at 16.6 to 25 cm/s, permitting the recording of eight to 13 consecutive steps comprised of three trials after a uniform gait for analysis of gait features.

#### Swimming test

Swim testing was performed in a rectangular plexiglass chamber (150 × 14.5 × 40 cm) with tap water maintained at 28°C with a heater at approximately 25 cm in depth for evaluation of aberrant posture involving clonus and spasticity during swimming in a contusive SCI as described previously.^41^ Briefly, the assessment was conducted in 10 runs at a 60 cm distance at the endpoint assessments in the early disuse group (n = 15) and the control group (n = 8) to assess the frequency of spastic posture that was characterized by typical aberrant posture involving stretched hindlimbs with unstable dorsi/ventro-flexed trunk, and/or whirled/erected tail position while swimming.

#### Electrophysiological testing

Terminal Hoffmann’s reflex (H-reflex) testing was performed as described previously.^41^ Briefly, the H-reflex was measured in the plantar interosseous muscle of the early disuse group and the control group (each n = 10) under sedation with chloral hydrate (2.5 g/kg, intraperitoneal injection, Sigma-Aldrich, St. Louis, MO) at the endpoint assessments. We tested the stimulus RDD of the H-reflex with the fixed stimulus intensity at 0.1, 0.5, 1, and 2 Hz in a randomized order across subjects. Peak-to-peak amplitudes of the H-reflex and M-wave were measured by obtaining 10-30 stimuli without the first stimuli.

Interlimb reflex testing was measured in the medial gastrocnemius muscle ipsilateral to the stimulated forepaw of the early disuse group and the control group (each n = 9) under sedation from pre-injury, 3 days post-injury and then once a week including just before/after suspension. Stimulation was fixed as 500 Hz of a 5-train pulse (each 1 ms duration) at the intensity of 1.0 mA (200 V) delivered percutaneously, and subjects received 10 to 15 trains of stimulation constantly with an inter-train interval of 3 s. The reflex response was analyzed for integrated electromyography area in two distinct periods, the early component (10-20 ms) and the late component (20-50 ms) of the interlimb reflex.

#### Synaptoneurosome biochemical analysis

Spinal cord ventral synapses were assayed via subcellular fractionation, protein assay, polyacrylamide gel electrophoresis, multiplexed immunoblotting, and quantitative near-infrared densitometric as described previously.^12^^,^^50^^,^^81^^,^^95^ Briefly, subjects (early disuse, n = 18; delayed disuse, n = 5; control, n = 22) were deeply anesthetized with pentobarbital sodium (100 mg/kg, intraperitoneal injection), decapitated, and spinal cords were harvested by rapid fluid expulsion with ice-cold PBS, immediately fresh-frozen, and stored at −80°C for later analysis. Thawed lumbar enlargements were dissected, and the ventral section was isolated sagittally and homogenized by glass-on-glass dounce homogenizer with ice-cold homogenization buffer (10 mM Tris, 300 mM sucrose, pH 7.5) containing protease inhibitor and phosphatase inhibitors. Sucrose gradient centrifugation at 5,000 relative centrifugal force (rcf) for 5 min at 4°C generated supernatant (S1) and the nuclear pellet (P1) fractions. Centrifugation of S1 at 13,000 rcf for 30 min yielded a synaptoneurosomal fraction (P2) and a cytosolic fraction (S2).

Protein concentrations were quantified using bicinchoninic acid assay, then each sample was diluted 1:2 with Laemmli sample buffer. Polyacrylamide gel electrophoresis was performed by loading 10 μg of protein per sample into an individual lane of a precast 10-20% Tris-HCl polyacrylamide gel with three independent replications according to a randomized counterbalancing fashion blindly.^96^^,^^97^ Protein was transferred to a nitrocellulose membrane, blocked, and probed with primary antibodies: rabbit polyclonal anti-GluA1 (1:200; Millipore, Billerica, MA; Cat# AB1504, RRID: AB_2113602), rabbit polyclonal anti-GluA2 (1:200; Millipore; AB1768-25UG, RRID: AB_2247874), rabbit monoclonal anti-pS831-GluA1 (1:200; Millipore; Cat# 04-823, RRID: AB_1977218), rabbit polyclonal anti-pS880-GluA2 (1:200; Millipore; Cat# 07-294, RRID: AB_568822), and mouse monoclonal anti-β-Actin as a loading control (1:1500; BD Biosciences; Cat# 612657, RRID: AB_399901) in blocking buffer. After washing with TBST, membranes were incubated with fluorescent secondary antibodies: goat anti-rabbit IRDye 680RD (1:30,000; LI-COR Biosciences; Cat# 925-68071, RRID: AB_2721181) and goat anti-mouse IRDye 800CW (1:30,000; LI-COR Biosciences; Cat# 926-32210, RRID: AB_621842). The membrane was immediately scanned using a 680 nm or 800 nm laser on the Odyssey Infrared Imaging System (LI-COR Biosciences, Lincoln, NE). Linear intensity detection of each fluorescently labeled protein band was quantified, and then rigorous statistical analysis of the normalized densitometry was performed using the established approach.

#### Spinal cord immunohistochemistry and automated confocal quantification

Spinal cord tissue preparation, immunohistochemistry, confocal image acquisition, automated image analysis, and rigorous statistical analyses were performed for quantitative immunohistochemistry as described previously.^12^^,^^50^^,^^95^ Briefly, all animal subjects (early disuse, n = 5; control, n = 6) were transcardially perfused with PBS, followed by 4% PFA under deep anesthesia. The lumbar enlargement was collected and embedded in OCT after overnight post-fixation in 4% PFA, and then immunofluorescence was performed on 20-μm-thick transverse frozen sections. Tissue sections were incubated with primary antibodies: rabbit monoclonal anti-GluA1 (1:200; Millipore; Cat# 04-855; RRID: AB_1977216), rabbit polyclonal anti-GluA2 (1:200; Millipore; Cat# AB1768-25UG; RRID: AB_2247874), and mouse monoclonal anti-Synaptophysin (1:200; Millipore; Cat# MAB5258-50UG; RRID: AB_95187) overnight at room temperature and then incubated with fluorescent secondary antibody: goat anti-rabbit IgG Alexa Fluor 488 (1:100; Thermo Fisher Scientific; Cat# A-11034; RRID: AB_2576217) and goat anti-mouse IgG Alexa Fluor 647 (1:100; Thermo Fisher Scientific; Cat# A-21236; RRID: AB_2535805), followed by coverslipped with mounting medium containing DAPI. Large ventral neurons (>40 μm diameter; presumptive motorneurons) were randomly selected on the location of the x, and y-axis using a Nikon spinning disk confocal system through a 650 nm filter for presynaptic synaptophysin and a 490 nm filter for postsynaptic AMPAR subunits GluA1 and GluA2 according to the established automated spatial sampling protocol (Figures 4A–4E). Confocal z-axis stack images were acquired with 0.25 μm step size through the neuron and then deblurred using 3-D blind iterative deconvolution. Fluorescently labeled punctum of synaptophysin, total AMPARs, and synaptic colocalized AMPARs on the neuropil of motorneurons were quantified using the custom-designed automated image analysis scripts. Fluorescently labeled extrasynaptic and synaptic AMPAR puncta on the plasma membrane of motorneurons were quantified in the single optical planes with the highest colocalization using the different scripts.

### Quantification and statistical analysis

#### Quantitative gait analysis

Circular phase diagrams were used as a means to assess the phase relationship between foot contact with the ground of an objective limb and the reference limb with respect to another within a gait cycle. Measures included the phase values (range from 0 to one), the radius vector (r) value reflecting the length of the vector that indicates the dispersion level of interlimb coordination (0 equal to high dispersion, and one equal to low dispersion), and the mean phase value reflecting the direction of the vector that suggests the phase variability on the basis of the consistency of 1:1 forelimb and hindlimb coordination.^98^^,^^99^^,^^100^^,^^101^ Plotted phase values indicate the timing of consecutive foot contacts of an objective limb on the reference limb per trial, and the gait cycle assessment was quantified with four types of interlimb coordination: contralateral forelimbs/hindlimbs and diagonal/ipsilateral forelimb-hindlimb (Figures 1 and S4). To assess the frequency of overstepping the range of mean ± 2 standard deviation (SD) of control in the phase value also suggests the dispersion level of interlimb coordination. All animals were trained a week prior to the assessment, and trials that showed less than 8 consecutive steps during ambulation were excluded from the gait analysis data.

#### Quantitative swimming assessment

The number of spastic postural events during swimming was counted for a frequency rate, and the peak timing was analyzed statistically by the cumulative frequency distribution. All data was normalized to the number of trials representing aberrant posture while swimming. Runs in which animals failed to make the entire distance of the swimming alley or defecated/urinated during swimming were excluded from assessments.

#### Quantitative electrophysiological measurement

For H-reflex measurement, a bipolar multi-stainless steel wire electrode was inserted subcutaneously on the plantaris muscle surface of the hind paw, and a ground electrode was placed on the surface of the tail (Figure 2E). Body temperature was monitored rectally and maintained at 37°C with a heat lamp and pad under sedation. Electromyography (EMG) was amplified and filtered with a bandwidth of 15-10,000 Hz with a bioamplifier (NEC Biotop 6R12; Nihon Kohden, Tokyo, Japan) and then converted to digital data with a sampling rate of 20 kHz with an AD converter (Micro 1401; Cambridge Electronic Design Ltd., Cambridge, UK) and stored for off-line analysis. 1 ms of rectangular electrical pulses were delivered via cuff electrodes mounted on the lateral plantar nerves of the hindlimb using a constant current stimulator (DS7A; Digitimer Ltd., Welwyn Garden City, UK). In the beginning, stimulation was given at the frequency of 0.1 Hz with a subthreshold stimulus intensity. Then, stimulus intensity was increased to acquire an observable H-reflex with as small M-response as possible (27.5 ± 22.8% of the maximum M-response) and maximum M-response.

Chronic EMG recordings were implemented through the multi-stainless steel wire electrodes implanted into the medial gastrocnemius muscle for interlimb reflex measurement (Figure 2I). Trains of electrical stimulation were subcutaneously delivered to the dorsal surface of the forepaw using the stimulator. Train pulses consist of 5 rectangular pulses, each 1 ms in duration, with an inter-pulse interval of 0.5 ms. EMG signals were rectified and averaged with respect to the first electric pulse.

#### Quantitative multiplexed near-infrared western blot

(1) Subcellular fractionation and spinal synaptoneurosomal enrichment

Spinal cord tissue preparation and subcellular fractionation were performed for western blotting as described previously.^12^^,^^50^^,^^81^^,^^95^ Briefly, spinal cords were harvested from the early disuse group (n = 18), the delayed disuse group (n = 5), and the control group (n = 22) by decapitation and rapid fluid expulsion with ice-cold phosphate-buffered saline (PBS, pH of 7.4) under deep anesthesia with pentobarbital sodium (100 mg/kg, intraperitoneal injection). Spinal cords were rapidly blocked into a 10 mm length of lumbar enlargement containing the L4-L5 spinal cord segments and rostral and caudal lengths. Samples were immediately snap-frozen in liquid nitrogen. The full extraction procedure from decapitation to snap freezing was timed with a stopwatch, and all samples were snap frozen in <5 min. Samples were stored at −80°C until the day of biochemical fractionation.

On the day of subcellular fractionation, snap-frozen spinal cords were thawed to −20°C on ice, and the ventral section was isolated from the lumbar enlargement using sagittal and horizontal cuts by a sterile scalpel blade. Ventral cords were homogenized using a glass-on-glass dounce homogenizer with a type B pestle with 200 μL of ice-cold homogenization buffer (10 mM Tris, 300 mM sucrose, pH 7.5) containing protease inhibitor (Complete Mini; Roche Diagnostics Corp., Indianapolis, IN) and phosphatase inhibitor cocktail (PhosSTOP; Roche Diagnostics Corp.). The resulting suspension was centrifuged at 5,000 relative centrifugal force (rcf) for 5 min at 4°C to pellet out nuclei and debris (P1). The supernatant (S1) was then centrifuged again at 13,000 rcf for 30 min at 4°C to yield the cytosomal fraction (S2) and synaptoneurosome/membrane-enriched fraction (P2) (Figure 3A). Fractions were resuspended with PBS containing a protease inhibitor and were stored at −80°C. Quantitative near-infrared western blot analysis was performed to identify spinal synaptoneurosomal enrichment in the P2 fraction (Figure 3B).

(2) Protein assay and SDS-PAGE

Protein concentration and quantitative multiplexed near-infrared western blotting were performed as previously described.^50^^,^^95^ Briefly, protein concentrations were quantified using bicinchoninic acid assay (BCA; Pierce, Rockford, Il) according to the manufacturer’s instructions and measured on a plate reader (GENios; Tecan, Männedorf, Switzerland). Sodium dodecyl sulfate-polyacrylamide gel electrophoresis (SDS-PAGE) of each individual sample (10 μg of total protein per sample) was performed by diluting the sample 1:2 with Laemmli sample buffer (62.5 mM Tris-HCl, pH 6.8, 25% glycerol, 2% SDS) containing 5% β-mercaptoethanol on ice, and then loading it into an individual lane of a precast 10-20% Tris-HCl polyacrylamide gel (Bio-Rad Laboratories, Inc., Hercules, CA). The left lane of the gel was loaded with 5 μL of Precision Plus Kaleidoscope protein ladder (Bio-Rad). The loaded gel was electrophoresed at 100V for one hour in SDS running buffer (25 mM Tris, 192 mM glycine, 0.1% SDS, pH 8.3; Bio-Rad).

Each subject was run in an individual lane, and each gel was run as a randomized counterbalancing block design containing equal numbers of subjects from each experimental group,^96^^,^^97^ performed by a biochemist blind to experimental conditions. All sample tubes were labeled using an arbitrary code and each gel had a pre-planned loading scheme determined *a priori* by a statistician uninvolved in the biochemical assay. Each sample was run in triplicate across multiple gels, and triplicate run (1-3) was recorded and entered into statistical models as a random effect, to directly assess bioassay variability.

(3) Quantitative multiplexed near-infrared immunoblotting and statistical densitometric analysis

Proteins were transferred onto a nitrocellulose membrane in a cold transfer buffer (25 mM Tris, 192 mM glycine, 20% methanol, pH 8.3). Membranes were blocked for one hour in Odyssey blocking buffer (LI-COR Biosciences) containing 0.1% Tween 20, then incubated overnight at 4°C with primary antibodies in Odyssey blocking buffer containing 0.05% Tween 20. To assess spinal synaptoneurosomal enrichment in all fractions, the membrane was probed with primary antibodies against the plasma membrane protein marker, mouse monoclonal anti-N-cadherin (1:800; BD Biosciences, San Jose, CA; Cat# 610920; RRID: AB_2077527) and mouse monoclonal anti-PSD-95 (1:2,000; Thermo Fisher Scientific, Waltham, MA; Cat# MA1-046; RRID: AB_2092361) with rabbit monoclonal anti-β-Actin as a loading control (1:1000; Cell Signaling Technology, Danvers, MA; Cat# 4970; RRID: AB_2223172). Following primary antibodies were used for assessing the level of excitatory neurotransmitter receptors in the ventral spinal cord: anti-GluA1, anti-GluA2, anti-pS831, anti-pS880, and anti-β-Actin as a loading control. Equal loading was confirmed by probing the same blots or reprobing the stripped blots^102^ with antibodies against β-Actin. Membranes were washed 4 × 5 min with Tris-buffered saline (TBS) containing 0.1% Tween 20 (TBST) and then incubated for one hour in the dark with fluorescently labeled secondary antibodies in Odyssey blocking buffer containing 0.2% Tween 20. Following fluorescent-labeled secondary antibody was used for each species of the primary antibody: goat anti-rabbit IRDye 680 and goat anti-mouse IRDye 800. After subsequently washing 4 × 5 min in TBST and 1 × 5 min in TBS, the membrane was immediately scanned for detecting each protein band using the corresponding 680 nm or 800 nm laser at a scanning appropriate intensity on the Odyssey Infrared Imaging System (LI-COR Biosciences).

All fluorescently labeled protein bands were quantified using our established protein dilution curve for each primary antibody to yield the linear range in which fluorescence was most highly correlated with changes in protein concentration using Odyssey Application Software Version 3.0 (LI-COR Biosciences). The optimal dilution and laser intensity at which protein concentration and fluorescence yielded the greatest linear correlation was established separately for each antibody and then held constant for the duration of the experiment (all R^2^ > 0.99) (Figures 3C and 3D). Each detected protein band fluorescently was quantified and normalized to median pixel density depending on the assessment of background fluorescence. All biochemistry was performed in a blinded, counterbalanced fashion and three independent replications. Normalized fluorescent densitometry results were pooled across runs using an advanced analytical workflow consisting of generalized linear mixed model regression with independent bioassay replication and horizontal well position entered as random effect variables. Full-length blots are shown in Figure S7.

#### Quantitative unbiased robotic confocal analysis

(1) Histological processing and immunofluorescence

Spinal cord tissue preparation and immunohistochemistry were performed for automated and unbiased high-resolution robotic confocal microscopy, as described previously.^12^^,^^50^^,^^95^ Briefly, rats were deeply anesthetized with pentobarbital sodium (100 mg/kg, intraperitoneal injection), given a bilateral thoracotomy, and perfused transcardially with ice-cold PBS followed by with 4% paraformaldehyde (PFA) solution. Lumbar enlargement containing the L4-L5 spinal cord segments was dissected out the vertebral column and post-fixed in 4% PFA solution for an additional 24 hours at 4°C prior to transfer to 20%, then 30% sucrose solution 24 hours at 4°C for cryoprotection. Spinal cord tissues were then frozen in liquid nitrogen and stored in a −80°C freezer until the day of tissue section. The tissue blocks were embedded in Tissue-Tek OCT compound (Sakura Finetek, Torrance, CA) before quick frozen on a dry ice chamber and then sectioned transversely into 20 μm thick slices on a cryostat (CM1950; Leica Biosystems GmbH, Nussloch, Germany). Tissue sections were divided into 10 adjacent sets so that a single set contained serial sections separated by >200 μm. After identifying the anatomical region using Luxol Fast Blue (LFB) staining, the tissue sections were blocked and permeabilized with 5% normal goat serum containing 0.3% triton-X 100 and then incubated overnight at room temperature with diluted primary antibodies of post-synaptic AMPAR subunit and pre-synaptic marker: anti-GluA1, anti-GluA2, and anti-Synaptophysin. For assessment of lesion pathology in the experimental animal model, the tissue sections of the thoracic spinal cord segments were incubated with diluted primary antibodies: rabbit polyclonal anti-NeuN (1:500; Millipore; Cat# ABN78; RRID: AB_10807945), mouse monoclonal anti-GFAP (1:500; Millipore; Cat# MAB360; RRID: AB_11212597), rabbit monoclonal anti-Vimentin (1:50; Cell Signaling Technology; Cat# 5741; RRID: AB_10695459), and rabbit polyclonal anti-GAP43 (1:500; Millipore; Cat# AB5220; RRID: AB_2107282). After washing slides with PBS repeatedly, the tissue sections were incubated for one hour in the dark at room temperature with fluorescent-labeled secondary antibody for each species of the primary antibody: Alexa Fluor 488 and Alexa Fluor 647. Slides were washed repeatedly with PBS and then coverslipped with Vectashield mounting medium with DAPI (Vector Laboratories, Burlingame, CA).

(2) Confocal image acquisition and 3-D blind iterative image deconvolution

All images were acquired at the UCSF Nikon Imaging Center using a Ti inverted microscope (Nikon Instruments, Melville, NY) with a spinning disk confocal scan system (CSU-22; Yokogawa Corporation, Sugar Land, TX) and a Plan Apo VC 63× oil immersion objective with 1.4 numerical aperture (Nikon Instruments), electron multiplying CCD camera running a custom instance of NIS elements imaging software (Nikon Instruments). Confocal images of large ventral horn neurons (>40 μm diameter; presumptive motorneurons) were selected in a semi-automated fashion using the established automated spatial sampling protocol (Figures 4A–4E).^12^^,^^50^ Briefly, a custom-designed macro generated an x,y coordinate list for high-resolution confocal data collection that the automated microscope stage uses as navigation points. Algorithmic inputs include key fiducial points: the ventral artery, the central canal, and lateral edges of the white matter in the spinal cord at the point of maximal diameter. All other data collection coordinates are generated *a priori* and then fed into ImageJ software (NIH, Bethesda, MD) and Micro-Manager software as automated regions of interest, ensuring that each cell body is sampled only once and that sampled cells are surrounded by 80 μm ‘guard zones’. This method ensures sample-window coverage of the ventral horns without overlap among sampled cells. The microscopist is only involved in centering the nucleus of each motorneuron in the field of view and setting the upper and lower limits of the z-series for confocal data collection blindly. Confocal z stacks of a motorneuron were taken with a z-step size of 0.25 μm along the rostrocaudal axis through a 650 nm filter for presynaptic synaptophysin and a 490 nm filter for postsynaptic AMPARs, respectively. AMPAR subunits GluA1 and GluA2 were assessed on adjacent slide sets from the same subjects.

Confocal z-series of ventral neurons were batch processed by 3-D blind iterative deconvolution using AutoQuant software (Media Cybernetics, Rockville, MD) to enhance the resolution beyond that achievable by spinning disk confocal alone. Deconvolution settings were optimized for optical setups of spinning disk confocal microscopy, photophoretic force, z-step size, and the optimal number of algorithm iterations on control tissue for each AMPAR subunit and then held constant throughout the duration of the experiment. A total of 164 cells with a total of 11,971 optical planes were assessed for GluA1, and a total of 138 cells with a total of 9,788 optical planes were assessed for GluA2 in the early disuse group (n = 5). A total of 163 cells with a total of 13,037 optical planes were assessed for GluA1, and a total of 156 cells with a total of 10,894 optical planes were assessed for GluA2 in the control group (n = 6).

(3) Automated quantification of AMPAR puncta on large ventral horn neurons

Quantification of synaptophysin-positive puncta, AMPAR subunits GluA1 and GluA2 levels was accomplished using a blinded and randomized analytical workflow of automated robotic high-resolution spinning disk confocal microscopy, followed by 3-D blind iterative deconvolution. AMPAR receptor levels were quantified from confocal z-series using MetaMorph image analysis software (Molecular Devices, San Jose, CA) with the custom-designed code.^12^^,^^50^ In brief, each z-stack was subjected to two separate waves of analysis. The first image processing pipeline quantified synaptic AMPAR subunits through the full dendritic field in the 3-D volume in a completely automated fashion by automatic thresholding and then quantifying positive puncta on the neuropil for synaptophysin, AMPARs, and synaptic colocalization for each optical plane through the neuron (Figures 4 and S8). The second image processing pipeline finds the optical plane with maximal colocalization and presents the synaptophysin channel to an experimentally blinded analyst, who then traces the membrane profile of the soma. The pipeline then expands this tracing to generate a 2 μm thick punch out of the plasma membrane, which sets then automatically thresholds, and the puncta area is quantified (Figure 5). Graphs and statistical results represent an analysis of >40,000 optical planes of large ventral neurons measured using these methods to quantify synaptic AMPAR changes in response to limb disuse. All multiple fluorescence images were converted for color-blind readers. All data acquisition and analyses were performed blind-to-condition.

#### Statistical analysis (univariate)

Statistical analyses were performed using SPSS Statistics for Windows, version 26.0 (IBM Corp., Armonk, NY) by biomedical statisticians uninvolved in data collection. The fundamental experimental design was a factorial repeated-measures design with a multi-dimensional (multivariate) endpoint assessment. Where appropriate, other random factors, nested factors, and covariates (including multiple time points, multiple bioassay results, multiple cells, and multiple optical planes) were included in the statistical model. Data were analyzed using the general linear model (GLM) command with random effects and covariates as appropriate (ANOVA, ANCOVA, random effects models, mixed designs with nested repeated measures). All bar graphs reflect estimated marginal means generated by SPSS. Error bars reflect the standard error of the mean (SEM) or the standard deviation (SD). The alpha level for all tests was set to 0.05. In all graphs, a statistically significant relationship among the groups for all outcome measures was indicated with a bar and an asterisk according to the following probabilities: ∗ = *P* ≤ 0.05, ∗∗ = *P* ≤ 0.01, ∗∗∗ = *P* ≤ 0.001. Complete statistical results of main effects, interactions, and post-hoc tests are reported in figure legends, and the sample size was determined by power calculations and effect sizes using eta-squared (η^2^) based on previous studies.^12^^,^^50^^,^^95^

#### Data analysis

Analysis of covariance (ANCOVA) was used to test for the main effect of the condition (i.e., early disuse, delayed disuse, and control) in western blot analysis. A separate model was used for each dependent variable (i.e., GluA1, GluA2, pS831, and pS880). The corresponding actin loading control (i.e., GluA1_Actin, GluA2_Actin, pS831_Actin, and pS880_Actin) was used as a covariate and replication (i.e., 1-3) was treated as a random factor. Tests of main effects were followed by pairwise comparisons of the control/experimental group's estimated marginal means (Bonferroni correction for multiple comparisons). The non-parametrical test (Wilcoxon signed-rank test) was used to compare two conditions (i.e., early disuse and control) in the swimming test. The sample size for each experiment was determined by power calculations and effect sizes using eta-squared (η^2^) based on previous studies (Tables S1 and S2).^12^^,^^50^^,^^95^

#### Machine learning: Non-linear principal component analysis (NLPCA)

NLPCA was harnessed to analyze multivariate patterns across all outcome measures, as described previously.^103^^,^^104^^,^^105^^,^^106^^,^^107^^,^^108^^,^^109^ Listwise deletion was used for the missing value in the NLPCA. Each subject was projected into the syndromic space by plotting their principal component (PC) scores for PC1-3. Each PC reflects an orthogonal linear combination of the variables that accounts for the maximum amount of the total variance in all outcome measures. The number of PCs was determined according to the criteria: 1) the Kaiser rule, retaining PCs with eigenvalues greater than 1; 2) the Cattell rule, retaining principal components above the elbow in the scree plot; 3) PC over-determination, retaining components with at least four PC loading values. PC scores were calculated using the regression method. All PC loading values were retained for PC interpretation. The validity of the PC loading pattern was assessed using GLM on the NLPCA-derived scores.
