# Supplementary material for: Disuse plasticity limits spinal cord injury recovery
Source: iScience. 2025 Mar 8;28(4):112180. doi: 10.1016/j.isci.2025.112180 (PMC11987634; doi:10.1016/j.isci.2025.112180)
Supplement: Document S1. Figures S1–S11 and Tables S1 and S2 [file mmc1.pdf]

iScience, Volume *28*

Supplemental information

Disuse plasticity limits

spinal cord injury recovery

Kazuhito Morioka, Toshiki Tazoe, J. Russell Huie, Kentaro Hayakawa, Rentaro Okazaki, Cristian F. Guandique, Carlos A. Almeida, Jenny Haefeli, Makoto Hamanoue, Takashi Endoh, Sakae Tanaka, Jacqueline C. Bresnahan, Michael S. Beattie, Toru Ogata, and Adam R. Ferguson

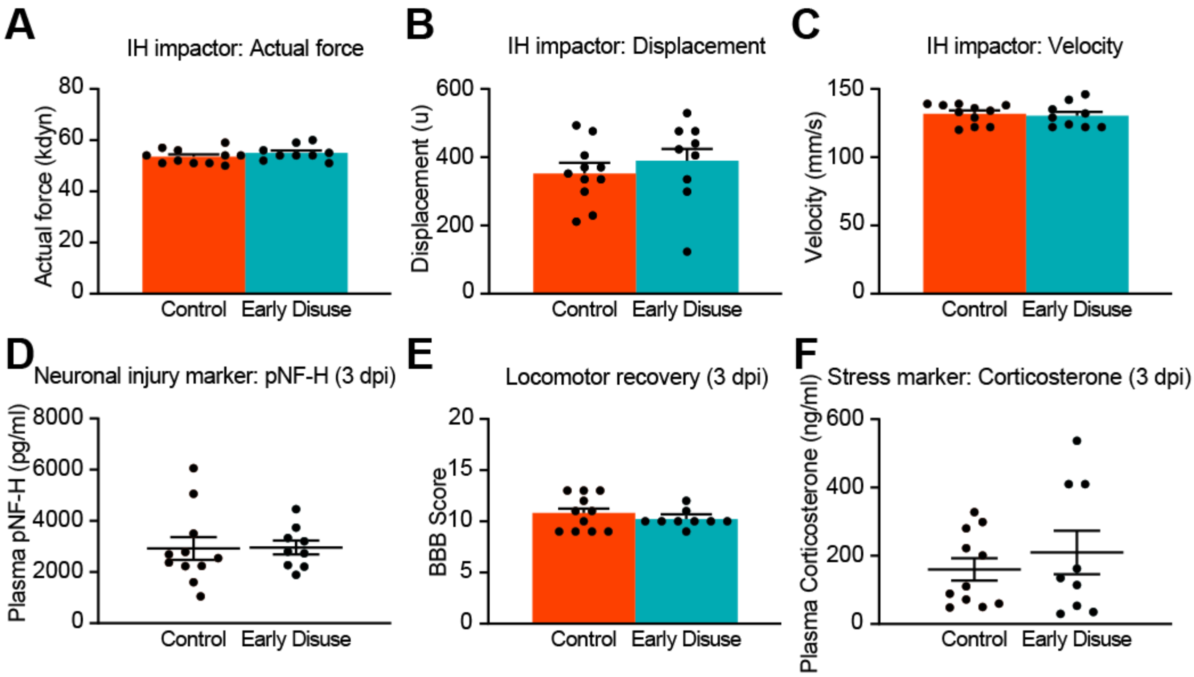

Figure S1 (Related to STAR Methods). Validation of early disuse after spinal cord injury animal model at 3 days post-injury.

(A-C) Profiles of the mild contusive thoracic SCI model represented no significant difference between the early disuse group (n = 9) and the control group (n = 11) in mean actual force (A), displacement (B), and velocity (C) of the IH impactor device (all $P > 0.05$). There is also no significant difference between both groups at 3 days post-injury in plasma levels of phosphorylated neurofilament heavy chain (pNF-H) for assessing the severity of SCI (D), the BBB open-field locomotor scoring for assessing recovery of locomotor function after SCI (E), and plasma levels of corticosterone for assessing the stress of suspension and/or SCI (all $P > 0.05$) (F). Statistically significant for $P < 0.05$ by One-Way Analysis of Variance (ANOVA). All data are shown as means ± standard error of the mean (SEM).

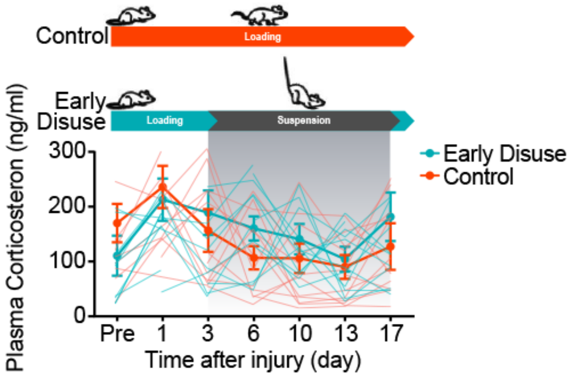

Figure S2 (Related to STAR Methods). Impact of early disuse after spinal cord injury on stress hormone levels.

To assess the stress levels from pre-injury to the terminal of suspension (week 2 post-injury) between the early disuse and control groups (each n = 10), repeated measurement of plasma corticosterone concentrations was performed from pre-injury to just after suspension. The early disuse group showed a time-dependent decrease during suspension, then increased again just after suspension/initial ambulatory reloading. Throughout all measurements, corticosterone levels of the early disuse group were higher than the control group, but there was no significant difference between both groups (all $P > 0.05$). Statistically significant for $P < 0.05$ by Two-Way ANOVA. All data are shown as means ± SEM.

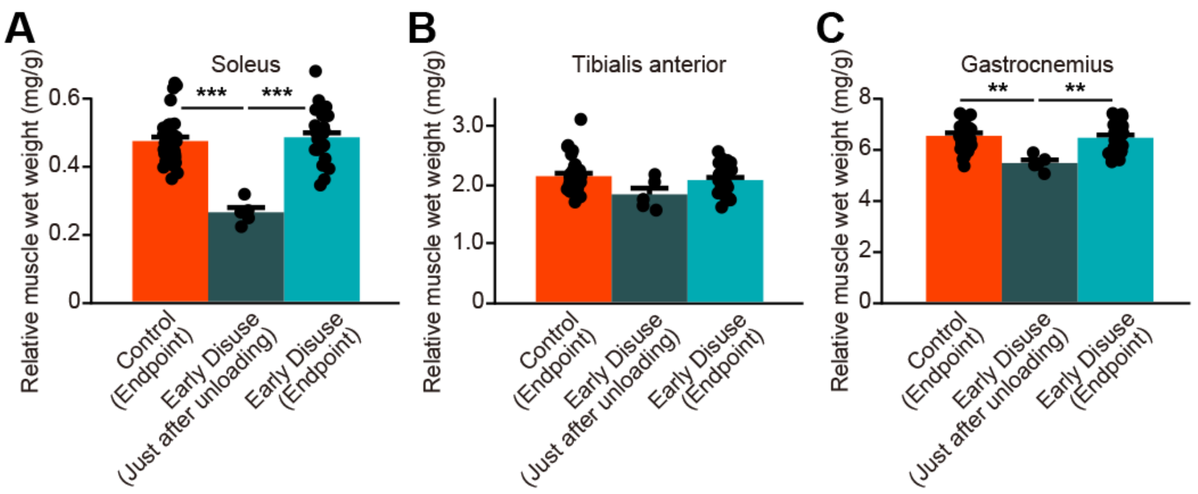

Figure S3 (Related to STAR Methods). Impact of early disuse after spinal cord injury on lower hindlimb muscle masses.

(A-C) To evaluate muscle alterations, individual muscle wet weight was normalized to the body weight in the early disuse group just after suspension (week 2 post-injury; n = 5, dark cyan) and after 6 weeks of reloading (week 8 post-injury; n = 28, cyan) compared to the control group at the endpoint assessment (week 8 post-injury; n = 34, orange). Early disuse caused a decrease in muscle weight immediately after suspension (week 2 post-injury) of two individual hindlimb muscles; soleus ($F(2,64) = 22.911$, ***$P = 0.000001$) (A) and gastrocnemius ($F(2,64) = 6.693$, **$P = 0.002$) (C), but no significant difference in tibialis anterior ($P > 0.05$) (B). In the later time points, early disuse did not significantly differ from the control (all $P > 0.05$). **$P < 0.01$, ***$P < 0.001$ by One-Way ANOVA with Tukey's post hoc test. All data are shown as means ± SEM. The average longitudinal body weight data is shown in Figure S11.

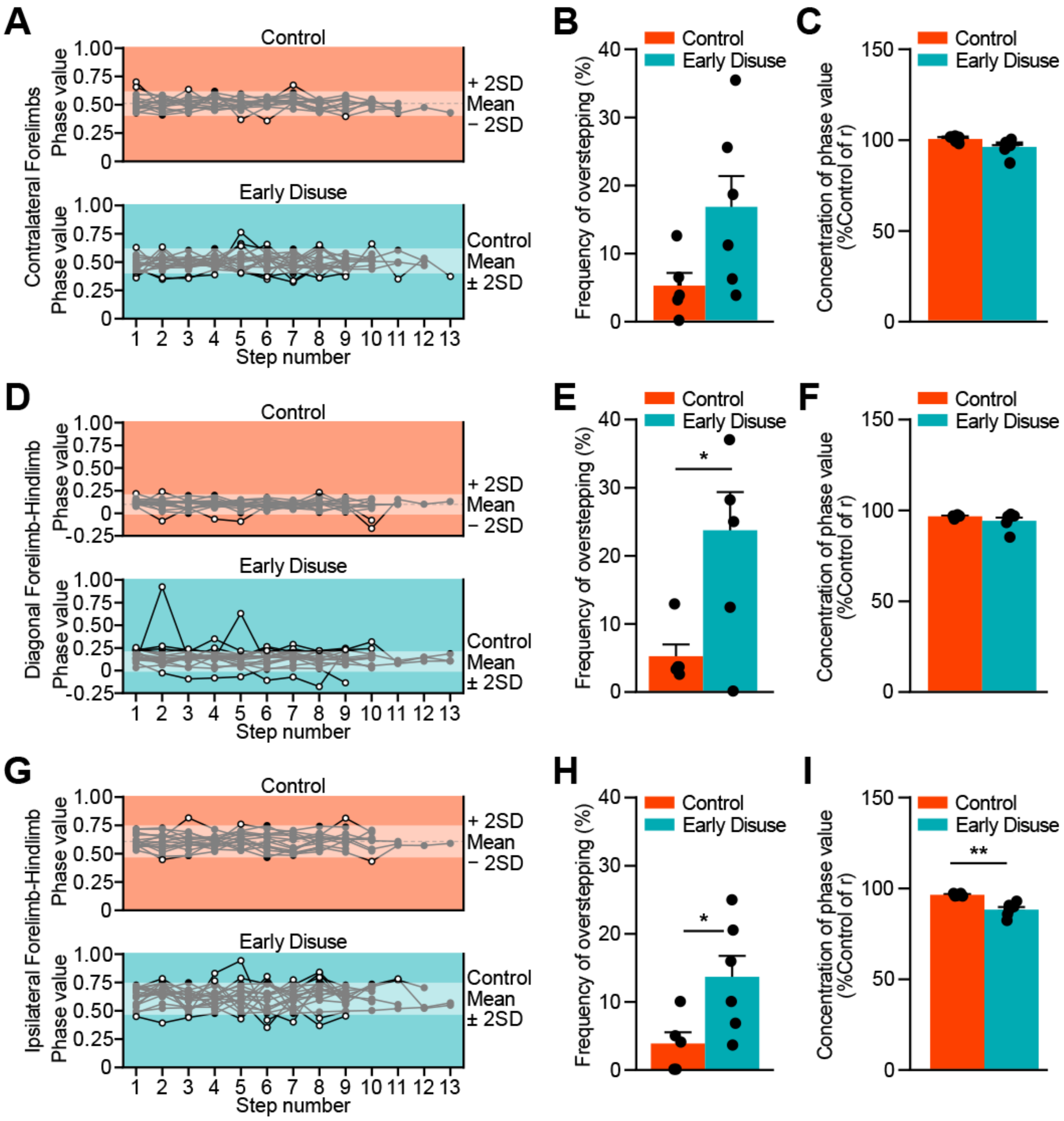

Figure S4 (Related to Figure 1). Additional gait profiles of interlimb coordination in early disuse after spinal cord injury animal model.

Four types of interlimb coordination during treadmill gait were examined in early disuse (n = 6) and control (n = 5) at week 8 post-injury: contralateral forelimbs (A-C), diagonal forelimb-hindlimb (D-F), ipsilateral forelimb-hindlimb (G-I), and contralateral hindlimbs (Figure 1J-1L). To assess the frequency of overstepping (white point) in the early disuse group (lower panel) compared with the range of mean ± 2 standard deviations (SD) of the control group (upper panel), the step sequence of an objective

limb relative to the reference limb was plotted per type of interlimb coordination (A, D, G, and Figure 1J). The quantitative frequency of overstepping showed a significant increase in the early disuse group at diagonal forelimb-hindlimb coordination (Effect of Early disuse condition: $F(1,9) = 6.94$, *$P = 0.027$; Effect of Trial: $F(2,18) = 1.369$, $P = 0.28$; Early disuse condition × Trial interaction: $F(2,18) = 0.514$, $P = 0.607$) (E), ipsilateral forelimb-hindlimb coordination (Effect of Early disuse condition: $F(1,9) = 5.773$, *$P = 0.04$; Effect of Trial: $F(2,18) = 0.901$, $P = 0.424$; Early disuse condition × Trial interaction: $F(2,18) = 3.644$, *$P = 0.047$) (H), and contralateral hindlimb coordination (Figure 1K). To evaluate the dispersion level of interlimb coordination (0 equals to high dispersion), the concentration of the phase value was measured. The early disuse group showed a significant decrease in ipsilateral forelimb-hindlimb coordination (Effect of Early disuse condition: $F(1,9) = 21.883$, **$P = 0.001$; Effect of Trial: $F(2,18) = 0.415$, $P = 0.666$; Early disuse condition × Trial interaction: $F(2,18) = 0.744$, $P = 0.489$) (I) as well as contralateral hindlimb coordination (Figure 1L), which is consistent with the results of the BBB open-field locomotor scoring (Figure 1A). *$P < 0.05$, **$P < 0.01$ by Two-Way ANOVA. All data are shown as means ± SEM.

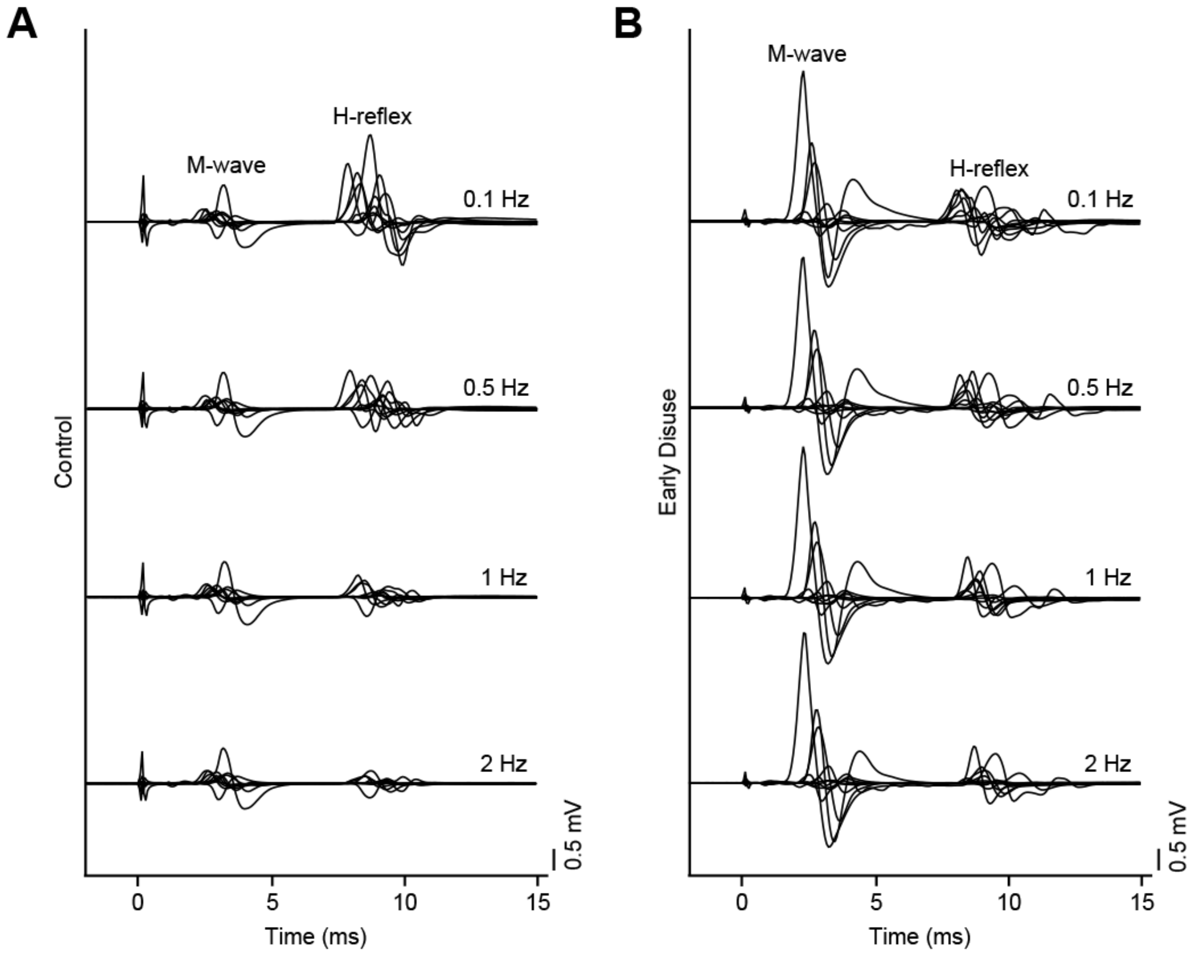

Figure S5 (Related to Figure 2). Electrophysiological profiles of the H-reflex testing in early disuse after spinal cord injury animal model.

The H-reflex testing was measured in the plantar interosseous muscle of the control group (A) and the early disuse group (B) (each n = 10) at week 8 post-injury. The mean amplitude of the M-wave responses was not significantly different across stimulus frequencies (all $P > 0.05$), whereas the mean amplitude of the H-reflex responses was significantly diminished in the early disuse group compared to the control group (Figure 2H). Statistically significant for $P < 0.05$ by Two-Way ANOVA. All data are shown as means ± SEM.

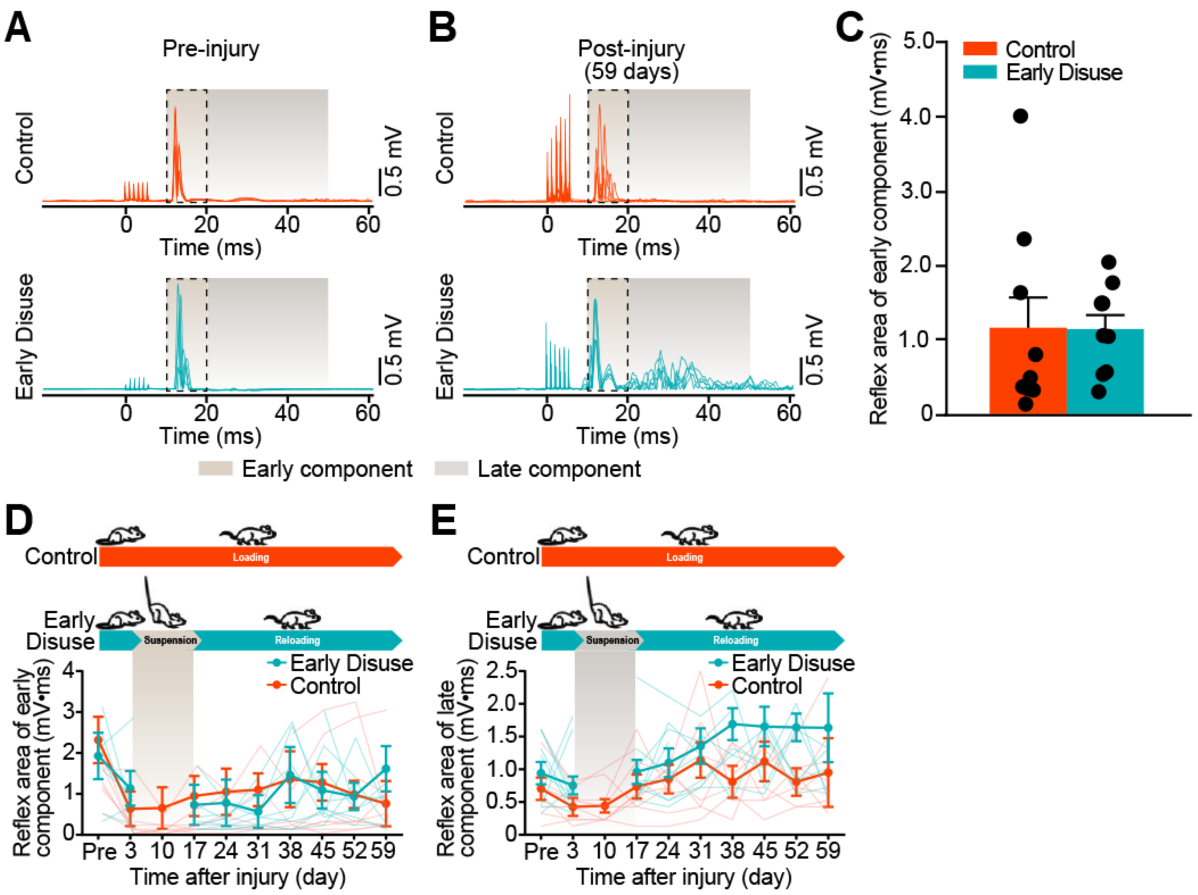

Figure S6 (Related to Figure 2). Additional electrophysiological profiles of the interlimb reflex testing in early disuse after spinal cord injury animal model. The Interlimb reflex testing was measured in the medial gastrocnemius muscle ipsilateral to the stimulated forepaw in the early disuse and control groups (each n = 9). Both groups demonstrated no differences at the early component (10-20 ms, brown) of the interlimb reflex in pre-injury (A) and at week 8 post-injury (B). (C) Quantitation revealed no significant main effect of early disuse condition on the early component of the interlimb reflex (all $P > 0.05$) but a significant difference on the late component (20-50 ms, gray) (Figure 2L). Non-significant interaction of early disuse condition and time was observed in the early component (D) and the late component (E) of the reflex response (the integrated EMG area; all $P > 0.05$). Statistically significant for $P < 0.05$ by One-Way or Two-Way ANOVA. All data are shown as means ± SEM.

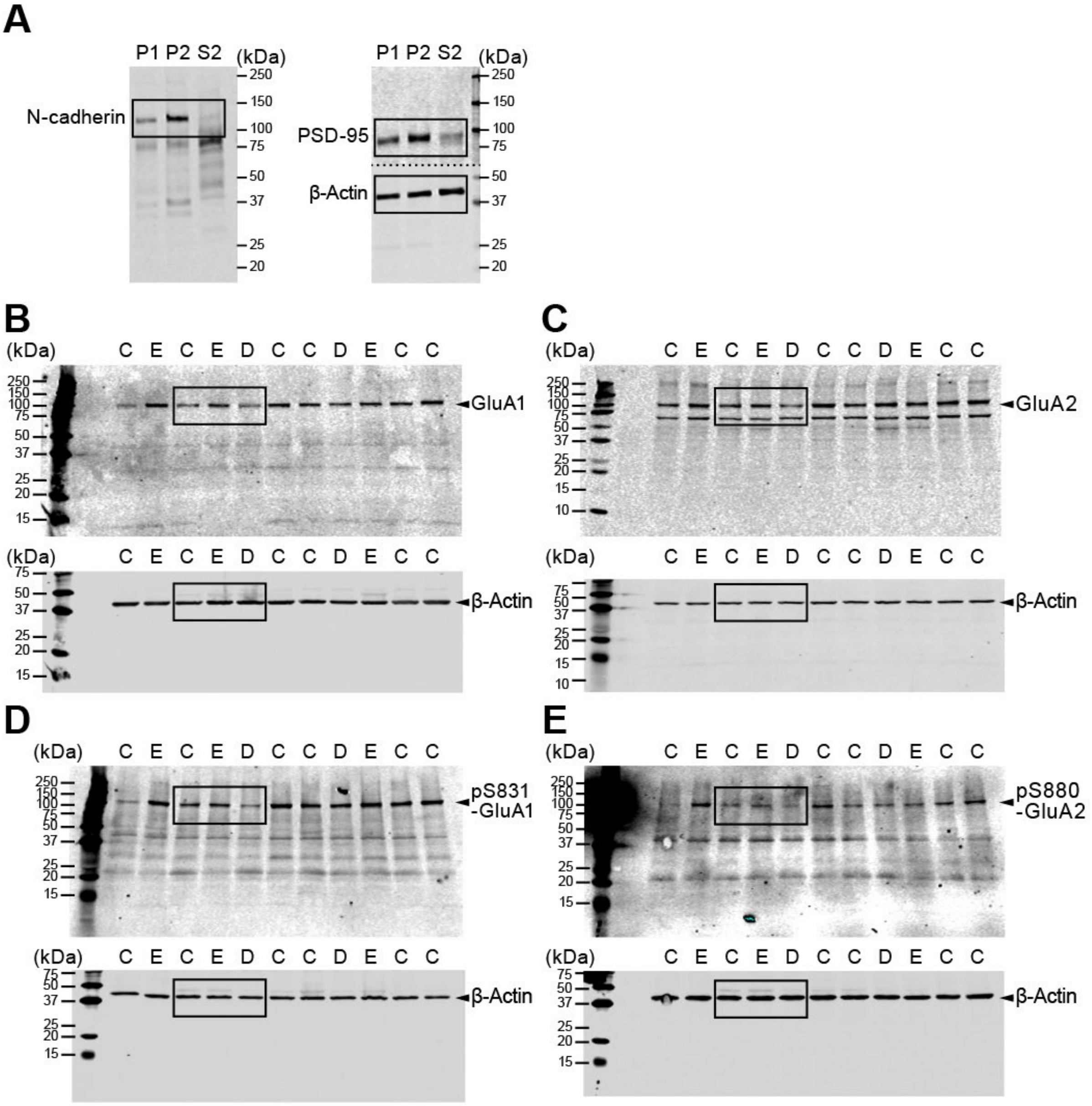

Figure S7 (Related to Figure 3). Immunobiochemical profile of synaptoneurosomal protein assay.

A full-length blot image of plasma membrane enrichment was detected in the P2 fraction of the lumbar ventral spinal cord with N-cadherin (A) (right panel). Synaptoneurosomal enrichment was characterized by PSD-95 with β-Actin serving as a loading control (left panel) within the same blots after stripping and reprobing (P1 = nuclear fraction; S2 = cytosolic fraction; see Figure 3). Representative full-length images of AMPAR expression in the synaptoneurosome and plasma membrane-enriched fractions (P2) were represented in upper panels of GluA1 (B),

GluA2 (C), GluA1 phosphorylation at PKC/CamKII site pS831 (pS831-GluA1) (D), and GluA2 PKC/CamKII target pS880 (pS880-GluA2) (E) with β-Actin as a loading control in each lower panel. All biochemical and western blot analyses were performed independently by three replications per sample (a total of 12 membranes for a total of 45 samples per probe). The experimental condition is indicated at the top of the image (C = Control; E = Early disuse; D = Delayed disuse). Representative adjacent images of the western blot in Figure 3 were highlighted with black boxes. All immunoblotting experiments were designed within the linear detection range for each analyte, and gels were run in a randomized block design, counterbalancing by location relative to the edge across the gels with blinding-to-condition for the rigorous statistical densitometric analysis of quantitative multiplexed near-infrared western blot.

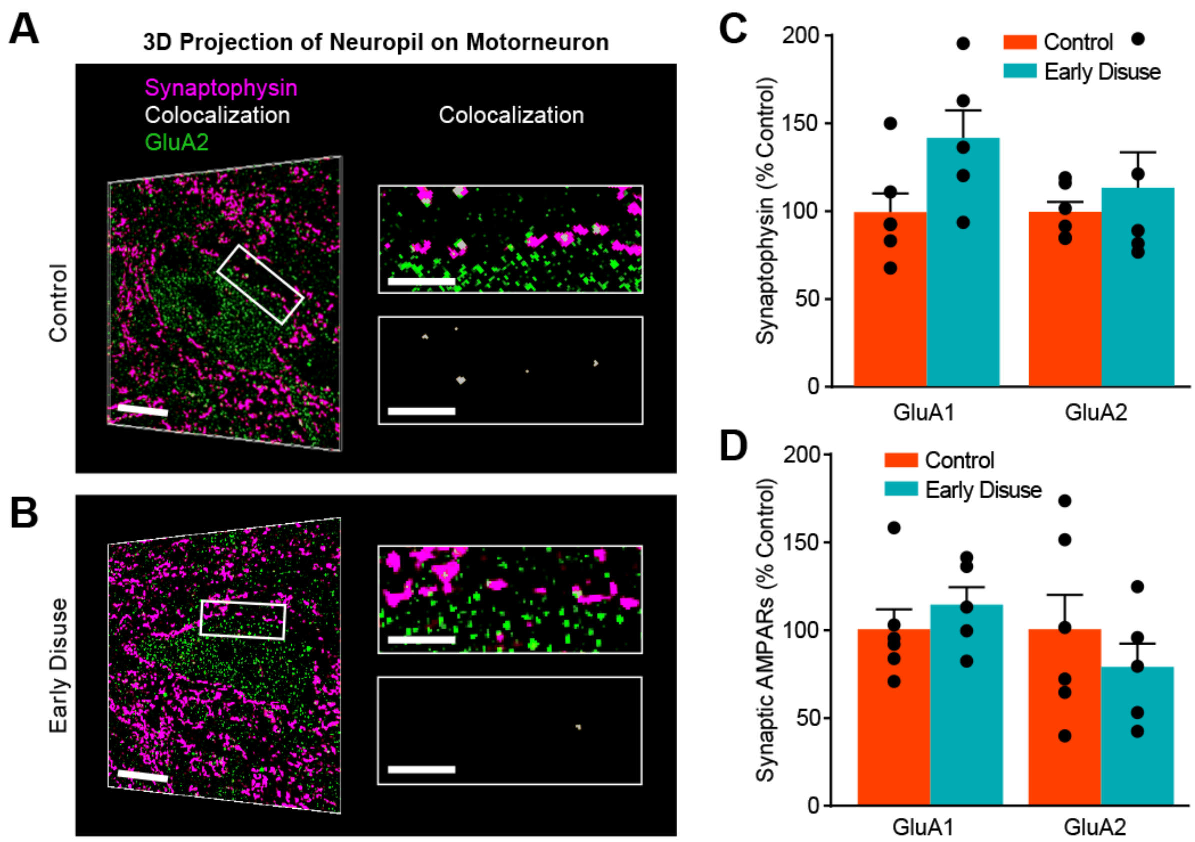

Figure S8 (Related to Figure 4). Immunohistochemical profile of synaptic colocalized GluA2 puncta on ventral horn neurons.

To assess synaptic levels of GluA2 on somata of large ventral horn neurons, the total expression of synaptic colocalized GluA2 puncta was quantified in the early disuse group (n = 5; total 138 cells, total 9,788 optical planes) and the control group (n = 6; total 156 cells, total 10,894 optical planes) using the established approach. Representative merged 3-D confocal images of large ventral horn neurons showed presynaptic synaptophysin (magenta) and postsynaptic GluA2 (green) on the somata with surrounding dendrites in the control subject (A) and the early disuse subject (B) (left panel). The enlarged image of the boxed region from each (A) and (B) (left panel) demonstrates relatively few synaptic colocalized GluA2 puncta (lower right panel, white) within the merge image (upper right panel) in the control subject and the early disuse subject, respectively. Random effects ANOVA controlling for non-independence of within-subject and within-section variability confirmed no significant

difference between both groups in synaptic colocalization of GluA2 (Effect of Early disuse condition: $F(1,9) = 0.074$, $P = 0.792$) (Figure 4H). There was also a non-significantly difference between both groups in presynaptic synaptophysin (Effect of Early disuse condition on GluA1: $F(1,9) = 4.299$, $P = 0.068$; Effect of Early disuse condition on GluA2: $F(1,9) = 0.408$, $P = 0.539$) (C) and postsynaptic AMPAR subunit (Effect of Early disuse condition on GluA1: $F(1,9) = 0.683$, $P = 0.43$; Effect of Early disuse condition on GluA2: $F(1,9) = 0.624$, $P = 0.45$) (D). Statistically significant for $P < 0.05$ by One-Way ANOVA. All data are shown as means ± SEM. Scale bars in the lower magnification images represent 20 μm (left panel), and the scale bar in the higher magnification image represents 5 μm (right panel).

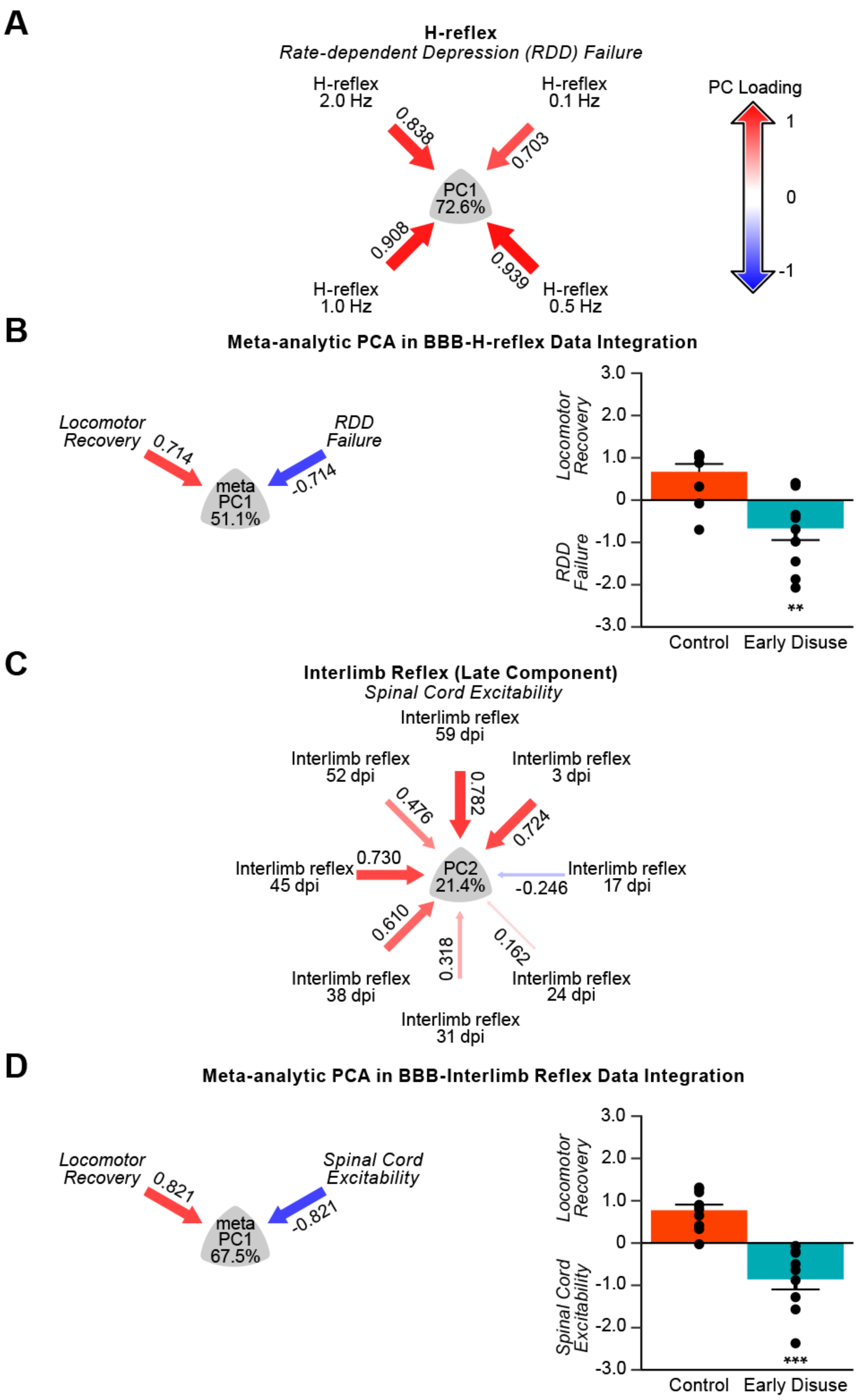

Figure S9 (Related to Figure 6). The robust multidimensional interactions

between hyper-excitability of spinal reflex circuits and impaired locomotor recovery in chronic spinal cord injury.

To identify the association between time-dependent locomotor recovery and chronic reflex hyper-excitability, two types of nonlinear meta-principal component analysis (NLPCA) were performed using the manifold learning approach. (A) PC loading pattern of diminished rate-dependent depression (RDD) of H-reflex accounted for 72.6% of total variance with high loading on PC1 outcome NLPCA of 'RDD Failure.' (B) Meta-analytic PCA in BBB-H-reflex data integration ($metaPCA_{H\text{-}reflex}$) of 'RDD Failure' and 'Locomotor Recovery' PC scores (Figure 6A) supported the hypothesis that chronic reflex hyper-excitability predicts the persistent locomotor impairments (left panel, 51.1% of total variance) and early disuse condition performs worse than control condition (right panel, Effect of Early disuse condition: $F(1,18) = 14.5$, $^{**}P = 0.001$). (C) PC loading pattern of enhanced responses of the late component of Interlimb reflex accounted for 21.4% of total variance with high loading on PC2 outcome NLPCA of 'Spinal Cord Excitability.' (D) Meta-analytic PCA in BBB-Interlimb reflex data integration ($metaPCA_{Interlimb\ reflex}$) of 'Spinal Cord Excitability' and 'Locomotor Recovery' PC scores supported the hypothesis that hyper-excitability of spinal reflex circuits predicts time-dependent locomotor recovery (left panel, 67.5% of total variance) and early disuse condition performs worse than control condition over time (right panel, Effect of Early disuse condition: $F(1,17) = 34.04$, $^{***}P = 0.00002$). $^{**}P < 0.01$, $^{***}P < 0.001$ by One-Way ANOVA. All data are shown as means ± SEM.

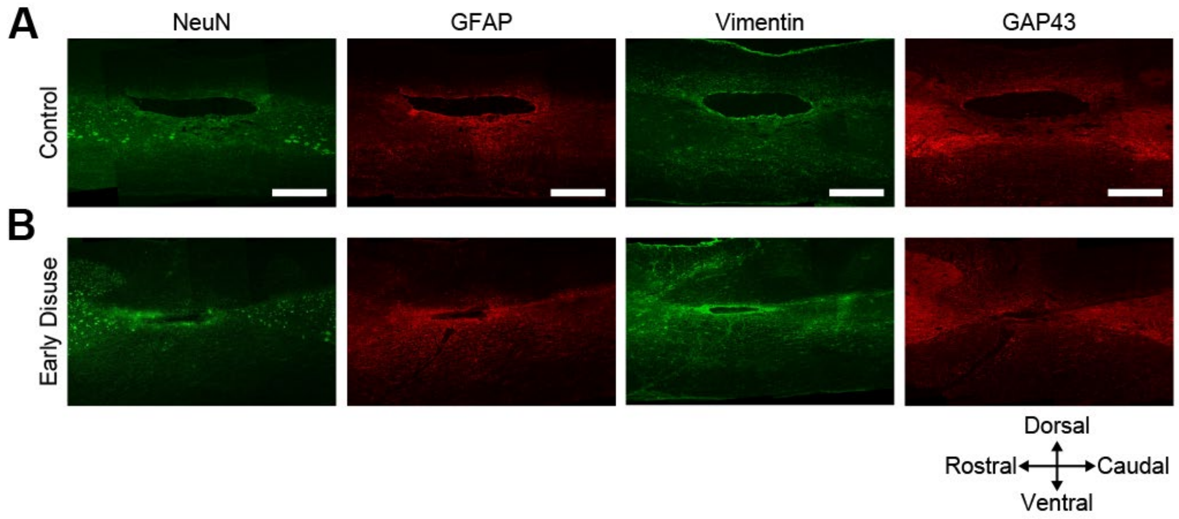

Figure S10 (Related to STAR Methods). Immunohistochemical profile of lesion pathology in early disuse after spinal cord injury animal model.

An overview of fluorescent immunolabelling with NeuN (green), GFAP (red), Vimentin (green), and GAP43 (red) in a longitudinal section demonstrates the thoracic lesion by the mild contusive injury in the dorsal region of the injured spinal cord at 59 days post-injury in the control subject (A) and the early disuse subject (B). Scale bars represent 500 μm.

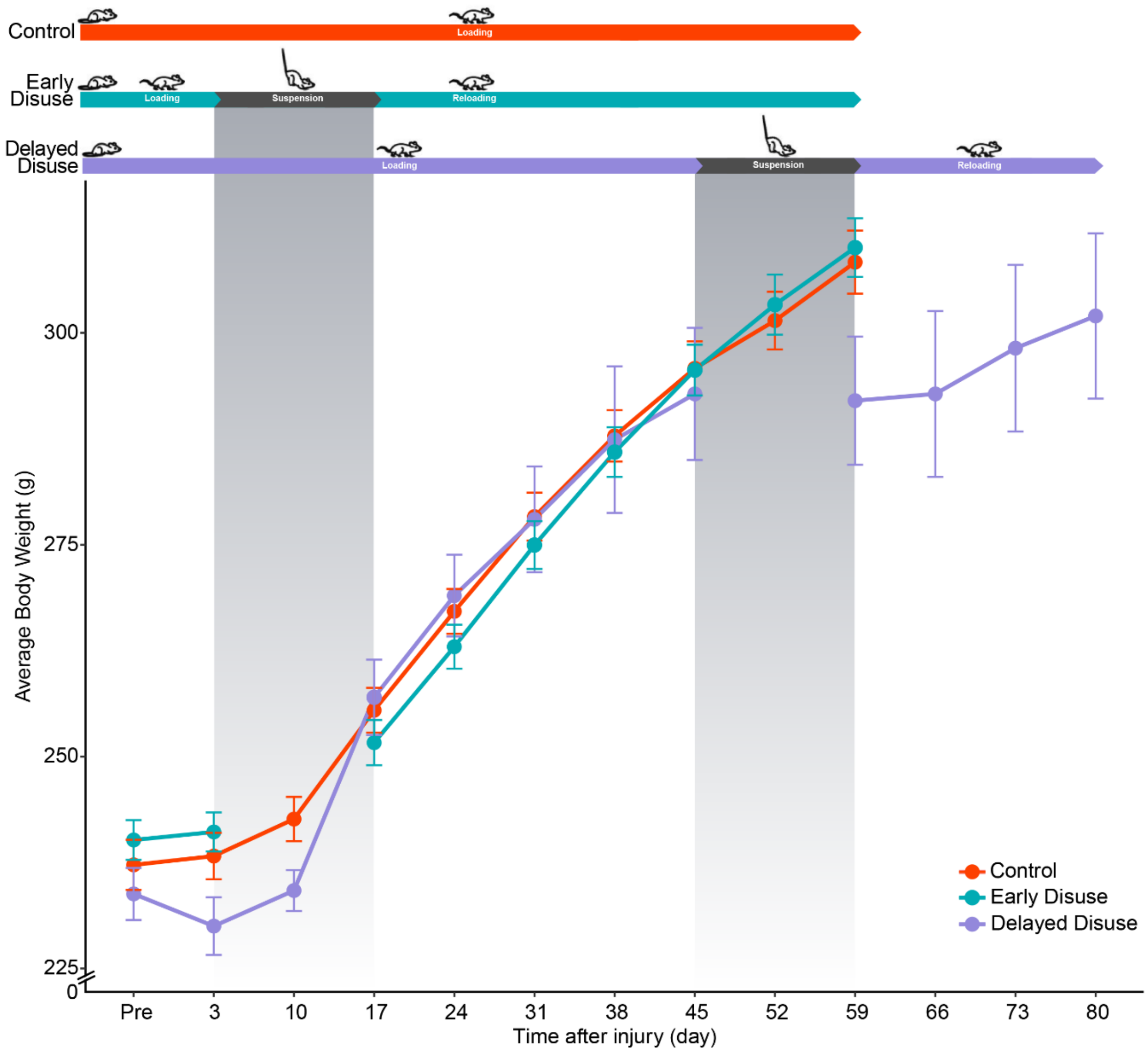

Figure S11 (Related to STAR Methods). Impact of early and delayed disuse after spinal cord injury on body weight over time.

An overview of average longitudinal body weight in the control group (normal loading throughout the assessment; n = 54, orange), the early disuse group (normal loading at 0-3 days post-injury, suspension at 3-17 days post-injury, reloading at 17-59 days post-injury; n = 63, cyan), and the delayed disuse group (normal loading at 0-45 days post-injury, suspension at 45-59 days post-injury, reloading at 59-80 days post-injury; n = 5, purple). All data are available at the Open Data for Spinal Cord Injury website (https://doi.org/10.34945/F52P4M).

Table S1. Sample size for each figure

	Number of Subjects per Experimental Group		
**Experiment**	**Control**	**Early Disuse**	**Delayed Disuse**
Open-field locomotor score (BBB) (Figure 1A-1C)	54	63	5
Gait analysis (Figure 1D-1L and Figure S4)	5	6	-
Swimming test (Figure 2A-2D)	8	15	-
H-reflex testing (Figure 2E-2H and Figure S5)	10	10	-
Interlimb reflex testing (Figure 2I-2L and Figure S6)	9	9	-
Synaptoneurosome biochemical analysis (Figure 3 and Figure S7)	22	18	5
Spinal cord immunohistochemistry and confocal analysis (Figure 4, Figure 5, and Figure S8)	6	5	-
Non-linear principal component analysis (Figure 6 & Figure S9)	54	63	5
Validation of injury severity (Figure S1)	11	9	-
Plasma conrticosterone concentration (Figure S2)	10	10	-
Hindlimb muscle mass (Figure S3)	34	28	-

Table S2. Sample size calculations

Figure	Data	Statistical test	Effect Size (η2)	Observed Power	Indication
Fig. 1A	Open-field locomotor score (BBB): Pre-SCI to 59 dpi	Two-way repeated measure ANOVA	0.728	1.000	***
Fig. 1C	Open-field locomotor score (BBB): Just before to after unloading	Two-way repeated measure ANOVA	0.505	1.000	***
Fig. 1K	Gait analysis: Frequency of overstepping on treadmill in contralateral hindlimbs	Two-way repeated measure ANOVA & UNIANOVA	0.635	0.940	**
Fig. 1L	Gait analysis: Concentration of the phase value on treadmill in contralateral hindlimbs	Two-way repeated measure ANOVA & UNIANOVA	0.758	0.997	***
Fig. 2C	Swimming test: Cumulative frequency distribution of developing spastic posture	Wilcoxon Signed-Rank Test	r = 0.600	0.960	**
Fig. 2D	Swimming test: Frequency of developing spastic posture	Two-way repeated measure ANOVA & UNIANOVA	0.498	0.991	***
Fig. 2H	H-reflex testing: H/M ratio in response to 0.1-2.0 HZ stimulation	Two-way repeated measure ANOVA & UNIANOVA	0.280	0.707	**
Fig. 2L	Interlimb reflex testing: Rectified and averaged EMG response in the late component	Two-way repeated measure ANOVA	0.277	0.642	*
Fig. 3E	Synaptoneurosome biochemical analysis: GluA1 protein expression	ANCOVA	0.876	0.969	**
	Synaptoneurosome biochemical analysis: GluA2 protein expression	ANCOVA	0.178	0.085	n.s.
Fig. 3F	Synaptoneurosome biochemical analysis: pS831-GluA1 protein expression	ANCOVA	0.940	0.988	**
	Synaptoneurosome biochemical analysis: pS880-GluA2 protein expression	ANCOVA	0.039	0.056	n.s.
Fig. 4H	Spinal cord immunohistochemistry and confocal analysis: GluA1 neuropil expression	UNIANOVA	0.472	0.715	*
	Spinal cord immunohistochemistry and confocal analysis: GluA2 neuropil expression	UNIANOVA	0.008	0.057	n.s.
Fig. 5M	Spinal cord immunohistochemistry and confocal analysis: GluA1 extrasynaptic membrane expression	UNIANOVA	0.490	0.745	*
	Spinal cord immunohistochemistry and confocal analysis: GluA2 extrasynaptic membrane expression	UNIANOVA	0.141	0.193	n.s.
Fig. 5N	Spinal cord immunohistochemistry and confocal analysis: GluA1 synaptic expression	UNIANOVA	0.396	0.582	*
	Spinal cord immunohistochemistry and confocal analysis: GluA2 synaptic expression	UNIANOVA	0.088	0.133	n.s.
Fig. 6D	Non-linear principal component analysis: BBB-Confocal data integrations	metaPCA	0.814	1.000	***
Fig. 6E	Non-linear principal component analysis: BBB-Western blot data integrations	metaPCA	0.310	0.971	***
Fig. S1A	Validation of injury severity: Observed force of IH impactor	UNIANOVA	0.064	0.183	n.s.
Fig. S1B	Validation of injury severity: Observed displacement of IH impactor	UNIANOVA	0.034	0.117	n.s.
Fig. S1C	Validation of injury severity: Observed velocity of IH impactor	UNIANOVA	0.008	0.065	n.s.
Fig. S1D	Validation of injury severity: pNF-H	UNIANOVA	0.000	0.050	n.s.
Fig. S1E	Validation of injury severity: Corticosterone 3dpi	UNIANOVA	0.012	0.072	n.s.
Fig. S1F	Validation of injury severity: BBB 3dpi	UNIANOVA	0.048	0.146	n.s.
Fig. S2	Plasma corticosterone concentration: Pre-SCI to 17 dpi	Two-way repeated measure ANOVA	0.003	0.055	n.s.
Fig. S3A	Hindlimb muscle mass: Soleus	UNIANOVA	0.417	1.000	***
Fig. S3B	Hindlimb muscle mass: Tibal anterior	UNIANOVA	0.085	0.560	n.s.
Fig. S3C	Hindlimb muscle mass: Gastrocnemius	UNIANOVA	0.173	0.903	**
Fig. S4B	Gait analysis: Frequency of overstepping on treadmill in contralateral forelimbs	Two-way repeated measure ANOVA & UNIANOVA	0.307	0.430	n.s.
Fig. S4C	Gait analysis: Concentration of the phase value on treadmill in contralateral forelimbs	Two-way repeated measure ANOVA & UNIANOVA	0.292	0.406	n.s.
Fig. S4E	Gait analysis: Frequency of overstepping on treadmill in diagonal forelimb hindlimb	Two-way repeated measure ANOVA & UNIANOVA	0.435	0.651	*
Fig. S4F	Gait analysis: Concentration of the phase value on treadmill in diagonal forelimb hindlimb	Two-way repeated measure ANOVA & UNIANOVA	0.127	0.176	n.s.
Fig. S4H	Gait analysis: Frequency of overstepping on treadmill in ipsilateral forelimb hindlimb	Two-way repeated measure ANOVA & UNIANOVA	0.391	0.573	*
Fig. S4I	Gait analysis: Concentration of the phase value on treadmill in ipsilateral forelimb hindlimb	Two-way repeated measure ANOVA & UNIANOVA	0.709	0.985	**
Fig. S6C	Interlimb reflex testing: Rectified and averaged EMG response in the early component	Two-way repeated measure ANOVA	0.000	0.050	n.s.
Fig. S8C	Spinal cord immunohistochemistry and confocal analysis: Synaptophysin expression in GluA1 neuropil	UNIANOVA	0.323	0.457	n.s.
	Spinal cord immunohistochemistry and confocal analysis: Synaptophysin expression in GluA2 neuropil	UNIANOVA	0.043	0.088	n.s.
Fig. S8D	Spinal cord immunohistochemistry and confocal analysis: Total GluA1 expression in neuropil	UNIANOVA	0.071	0.115	n.s.
	Spinal cord immunohistochemistry and confocal analysis: Total GluA2 expression in neuropil	UNIANOVA	0.065	0.109	n.s.
Fig. S9B	Non-linear principal component analysis: BBB-H-reflex data integrations	metaPCA	0.448	0.951	**
Fig. S9D	Non-linear principal component analysis: BBB-Interlimb reflex data integrations	metaPCA	0.666	1.000	***

Note. * = P ≤ 0.05, ** = P ≤ 0.01, *** = P ≤ 0.001; not significant (n.s.) = P > 0.05.
